# Systematic review of the epidemiology of acne vulgaris

**DOI:** 10.1038/s41598-020-62715-3

**Published:** 2020-04-01

**Authors:** Anna Hwee Sing Heng, Fook Tim Chew

**Affiliations:** Department of Biological Sciences, National University of Singapore, Singapore, Singapore

**Keywords:** Skin diseases, Risk factors, Epidemiology

## Abstract

A systematic review was conducted on epidemiology studies on acne obtained from a Web of Science search to study risk factors associated with acne presentation and severity. A strong association was observed between several risk factors – family history, age, BMI and skin type – and acne presentation or severity in multiple studies. The pooled odds ratio of 2.36 (95% CI 1.97–2.83) for overweight/obese BMI with reference to normal/underweight BMI and the pooled odds ratio of 2.91 (95% CI 2.58–3.28) for family history in parents with reference to no family history in parents demonstrate this strong association. In addition, a pooled odds ratio of 1.07 (95% CI 0.42–2.71) was obtained for sex (males with reference to females). However, the association between other factors, such as dietary factors and smoking, and acne presentation or severity was less clear, with inconsistent results between studies. Thus, further research is required to understand how these factors may influence the development and severity of acne. This study summarizes the potential factors that may affect the risk of acne presentation or severe acne and can help researchers and clinicians to understand the epidemiology of acne and severe acne. Furthermore, the findings can direct future acne research, with the hope of gaining insight into the pathophysiology of acne so as to develop effective acne treatments.

## Introduction

## Acne Epidemiology

The Global Burden of Disease Study 2010 found that acne vulgaris (henceforth acne) is the eight most common skin disease, with an estimated global prevalence (for all ages) of 9.38%^1^. In different countries and among different age groups, the prevalence of acne varies, with estimates ranging from 35% to close to 100% of adolescents having acne at some point^2^.

## Symptoms and Impacts of Acne

Acne patients typically present with comedones, papules and pustules^3^. Comedones can be subdivided into two types – open comedones (blackheads), which are clogged follicles with openings exposing its contents to the air, and closed comedones (white heads), which are clogged follicles without an opening^4^. Papules are raised lesions on the skin that are smaller than 1 cm in diameter while pustules are similar to papules but inflamed and filled with pus^4^. In patients with severe acne, nodules and cysts – inflamed, swollen lesions that are at least 5 mm large – may be present^3,4^. In addition, other symptoms such as the scars, erythema and hyperpigmentation may be observed in acne patients^4^.

On top of discomfort due to the clinical symptoms of acne, patients may experience other negative impacts. A study observed significantly higher unemployment rates among acne cases relative to controls, suggesting a correlation between acne and employment^5^. Further, acne has been found to adversely affect the social life^6^, self-esteem and body image of individuals and is often co-morbid with psychological disorders including depression and anxiety^3^. Additionally, acne is associated with substantial financial costs, with one study estimating that the cost of treating acne in Germany adds up to 400 million Euros annually^7^.

## Aim

This review aims to analyze the epidemiology of acne around the world and investigate the factors that significantly modify the risk of presenting the condition.

## Results and Discussion

### Epidemiology of acne

The 35 articles reviewed differed in study design, acne definition and severity grading systems, variables studied and population characteristics. Population characteristics such as age and sex differed between studies depending on the type of acne and variables the researchers were interested in. For instance, Wei *et al*.^8^ studied adolescent acne, Kaminsky, Florez-White, Bagatin and Arias^9^ studied adult acne, and Park, Kwon, Min, Yoon and Suh^10^ studied childhood acne. Similarly, some studies only studied the risk factors for acne in females^11^ while others only studied acne in males^12^. Sample sizes used also varied, ranging from 88 in Ismail, Manaf & Azizan^13^ to 27,083 in Klaz, Kochba, Shohat, Zarka & Brenner^14^. Further, some studies investigated the co-morbidities of acne, however, since this paper is not intended to provide a review of acne co-morbidities, they will not be discussed here.

The acne and severity grading systems used by the different studies is described in Table 1. Depending on the acne definition and severity grading system used, the resulting prevalence estimates differ. Despite the presence of objective symptoms of acne (such as the presentation of comedones, papules and/or pustules), dermatologists disagree about the minimal criteria that should be used to diagnose the condition^15^. Similarly, efforts to create a standardized grading system for acne severity have been unsuccessful and over 25 different systems are currently in use^16^. As such, different studies use different definitions and grading criteria, making it difficult to compare their results and derived prevalence estimates^3,16^. The prevalence estimates obtained are also influenced by other factors such as the sample size and country studied. A larger sample will result in a more representative prevalence estimate. Prevalence estimates ranged from 26.8% in a study conducted in Germany^17^ to 96% in a study conducted in Brazil^18^. A summary and description of the articles reviewed can be found in Table 1.

**Table 1:** Summarised descriptions of journal articles on acne published between 1999 and 2019.

Country	Sample (size, age)	Study design	Prevalence	Definition of acne	Parameters that differ between control and case	Parameters that differ between groups of different acne severity	Severity grading system	Acne grading system	Ref, date
**Cross-sectional design**									
Turkey, Eskisehir	2300 individuals aged 13–18 years	Cross-sectional, self-report questionnaire	60.7% of the 2230 participants (after exclusion of participants who did not answer at least 90% of questionnaire)	Clinical diagnosis by dermatologists	Significant risk factors: age, BMI, diet (fat, sugar intake, frequent intake of fast food, desserts) Significant protective factors: diet (fruit and vegetable intake) frequency of face washing per day (with tap water), living environment Insignificant factors: sex	Significant risk factors associated with increased acne severity: acne duration, age, BMI, living environment, sex, skin type (oily) Insignificant factors: family history	Pillsbury’s diagnostic criteria	Presence of any acne lesion	Aksu, *et al*., 2012
Pakistan, Quetta	1000 teenagers and young people	Cross-sectional, interview using a questionnaire	65% in teenagers and 28% in adults (overall prevalence not reported)	Self-reported acne	Significant protective factors: age, diet (non-spicy food intake) premenstrual stage, marital status, sex, skin type (dry, normal, oily)	N/A	N/A	Self-reported acne	Ali, *et al*.^50^
Romania, Tîrgu Mureș	148 high school students aged 16–20 years	Cross-sectional, self-report questionnaire	47.3% of high school students	Clinical diagnosis of acne vulgaris by a dermatologist	Significant risk factors: BMI, diet (carbonated drink, fat, white bread, sweets intake), family history (parents), smoking status Significant protective factors: diet (fish, fruits/vegetables intake) Insignificant factors: diet (dairy intake), irregular meals, lack of nutritional information, living environment	Significant risk factors associated with increased acne severity: BMI, diet (fat, sweets intake) Significant risk factors associated with decreased acne severity: diet (fruits/vegetables intake) Insignificant factors: diet (general, carbonated drink, dairy, fish, white bread intake), family history, lack of nutritional information, living environment, smoking status	Numbers and types of inflammatory and non-inflammatory acne lesions	Presence of any acne lesion	Al Hussein, *et al*., 2016
Brazil, São Paulo	452 students aged 10 to 17 years	Cross-sectional, self-report questionnaire	96% of students	Clinical diagnosis by 3 independent evaluators	Insignificant factors: family history (parents and relatives), parent’s educational level, skin colour	Significant risk factors associated with increased acne severity: age, family history (siblings), parent’s education level Insignificant factors: family history (first-degree relatives excluding siblings), race, sex, skin colour, smoking status	Numbers and types of inflammatory and non-inflammatory acne lesions	Presence of any acne lesion	Bagatin, *et al*., 2014
Brazil, Pelotas	2,201 males aged 18 years	Cross-sectional, self-report questionnaire	89.1% of males	Clinical diagnosis by a dermatologist	Significant protective factors: height (short), skin colour (light) Insignificant factors: BMI, diet (cheese, chocolate, low fat milk, whole milk, yoghurt intake), smoking status, years of education	Significant risk factors for inflammatory lesions only: height (tall), skin colour (light) Insignificant factors for inflammatory lesions only: BMI, diet (cheese, chocolate, low fat milk, whole milk and yoghurt intake), smoking status, years of education Significant risk factors for noninflammatory acne only: height (tall), skin type (dark), Insignificant factors for noninflammatory acne only: BMI, diet (cheese, chocolate, low fat milk, whole milk and yoghurt intake), smoking status, years of education, Significant risk factors for both inflammatory and noninflammatory acne: diet (yoghurt intake), height (tall), skin colour (light) Insignificant factors for both inflammatory and noninflammatory acne: BMI, diet (cheese, chocolate, low fat milk and whole milk intake), smoking status, years of education	Numbers and types of inflammatory and non-inflammatory acne lesions	Presence of any acne lesion	Duquia, *et al*., 2017
Iran, Tehran	1002 students aged 12–20 years	Cross-sectional, self-report questionnaire	93.2% of the students	Clinical diagnosis by dermatologists and general practitioners	N/A	Significant risk factors associated with increased acne severity: age, diet (chocolates/sweets, nuts, oily food intake), family history (parents and siblings), mental stress, number of family members with acne history, personal evaluation of skin oiliness, premenstrual phase, skin type Insignificant factors: age of menarche, diet (spicy food intake), fasting, frequency of face washing per day, physical exercise, regularity of menses, seasons of the year, sex, sleep duration, smoking status, sun exposure, travel to humid regions, use of cosmetics, winter skin	Global Alliance to Improve Outcomes in Acne	Global Alliance to Improve Outcomes in Acne	Ghodsi, Orawa, & Zouboulis^51^
Ghana, Greater Accra	1394 children aged 9 to 16	Cross-sectional, physical examination	N/A	Clinical diagnosis by dermatologists	Significant risk factors: age, BMI, living environment, sex	N/A	N/A	Physical examination, presence of at least six facial pustules or papulopustules	Hogewoning, *et al*., 2009
Latin America and Iberian Peninsula, 21 countries	1384 acne cases aged 25 to 60	Cross-sectional, self-report questionnaire	N/A	Clinical diagnosis by a dermatologist	N/A	Significant risk factors associated with increased acne severity: acanthosis nigricans, exposure to chemical substances, hirsutism, hyperseborrhea, onset of acne during adolescence, sex Significant risk factors associated with decreased acne severity: makeup use, Insignificant factors: age at menarche, alopecia, climate, diet, family history, onset of menopause, sun exposure, sunbed usage, tobacco use, use of contraceptives (hormonal), regular use of acne drugs	GILEA acne clasification	N/A	Kaminsky, Florez‐White, Bagatin, & Arias, 2019
Lithuania	1277 students aged 7–19 years	Cross-sectional, self-report questionnaire	82.9% of the 1229 participants who underwent clinical diagnosis	Clinical diagnosis by a dermatologist	Significant risk factors: age, BMI, family history (parents), onset of puberty, Insignificant factors: alcohol intake, diet (dairy, fast food, fish, fruits/vegetables, lemonade, meat, sweets intake), smoking status	Significant risk factors associated with decreased acne severity: BMI, Significant risk factors associated with mild and moderate/severe acne: family history (maternal acne, paternal acne and acne in both parents) Insignificant factors: onset of puberty, sex	Leeds revised acne grading system	Presence of any acne lesion	Karciauskiene, Valiukeviciene, Gollnick & Stang, 2014
Israel, Tel Aviv	27083 males aged 21–22 years	Cross-sectional, interviews	0.88% severe acne (prevalence of mild/moderate acne was not reported)	Clinical diagnosis by dermatologists	N/A	Significant risk factors associated with decreased acne severity: number of cigarettes smoked per day, smoking status	Kligman and Plewig grading and Leeds acne grading system	N/A	Klaz, Kochba, Shohat, Zarka & Brenner, 2006
South Korea, Seoul	693 elementary school students aged 7–12 years	Cross-sectional, self-report questionnaire	36.2% of elementary school students	Clinical diagnosis by dermatologists	Significant risk factors: age, BMI, diet (chocolates/sweets intake) Insignificant factors: diet (meat, pizza intake), number of face washings, sex, sleep duration, use of moisturiser,	Significant risk factors associated with increased acne severity: age	Severity grading was based on Lehmann *et al*. and the Leeds Revised Acne Grading System	Presence of any acne lesion	Park, Kwon, Min, Yoon, & Suh, 2015
Sri Lanka, Colombo	140 females aged 15–16	Cross-sectional, self report questionnaires	91.4% of individuals	Assessment by interviewers	N/A	Significant risk factors associated with increased acne severity: use of cosmetics	Grading scale for overall severity (GSOS)	N/A	Perera, Peiris, Pathmanathan, Mallawaarachchi, & Karunathilake^52^
Belgium, Antwerp	594 secondary school students aged 13 to 18	Cross-sectional, interviews	95.6% with at least one retentional acne lesion on the face	Clinical diagnosis by a dermatologist	Significant risk factors: sex Significant protective factors: number of cigarettes smoked per day (in females), smoking duration (in females), smoking status (in females) Insignificant factors: age of menarche, drug usage, physical exercise, multivitamin consumption, number of cigarettes smoked per day (in males), regularity of menses, smoking duration (in males), smoking status (in males), sunbed usage	Significant risk factors for mod/severe acne: drug usage (high usage of topical and/or systemic drugs to treat acne), sex (male) Significant protective factors for mod/severe acne: number of cigarettes smoked per day (in females), smoking duration (in females), smoking status (in females), use of contraceptives (oral)	ECLA (Echelle d’Evaluation Clinique des Lesions d’Acné) scale	>20 retentional and/or inflammatory acne lesions on the face	Rombouts, Nijsten & Lambert, 2006
Germany, Hamburg	896 individuals aged 1–87 years (median age = 42 years)	Cross-sectional, standardised interview	26.8% of participants	Clinical diagnosis by dermatologists	Significant risk factors: age, sex, smoking status, number of cigarettes smoked per day Insignificant factors: alcohol intake, socioeconomic status	Significant risk factors associated with increased acne severity: number of cigarettes smoked per day Insignificant factors: sex	Numbers and types of inflammatory and non-inflammatory acne lesions	Presence of any acne lesion	Schäfer, Nienhaus, Vieluf, Berger, & Ring, 2001
US	9417 individuals aged 0–17 years	Cross-sectional, self-report questionnaire	2.8% of the participants had severe acne	Self-reported acne	N/A	Significant risk factors associated with increased acne severity: age, educational level in the family at age 14–15, gastrointestinal conditions (reflux, abdominal pain, nausea, food allergy), number of children at age 16–17, psychological disorders (depression, anxiety, ADHD/ADD, insomnia), race at age 14–15, sex at age 11–13, sinopulmonary disorders (sinus infection, sore throat, asthma, lung disease excluding asthma, non-streptococcal pharyngitis), Insignificant factors: duration of residence in the US, gastrointestinal conditions (frequent diarrhoea, intermittent constipation), Hispanic origin, household income, place of birth (outside the US), psychological disorders (phobias), race at age 11–13 and 16–17, sinupulmonary disorders (tonsillitis, hay fever, respiratory allergy)	“Yes” response to the question ‘During the past 12 months, has (child) had severe acne?’	N/A	Silverberg & Silverberg, 2014
Singapore	1045 youths aged 13–19 years	Cross-sectional, self-report questionnaire	88% self-reported acne	Self-reported acne status, dermatologist diagnosis for individuals who reported that they had acne	N/A	Insignificant factors: age, family history, race, sex	Criteria defined by Lehmann *et al*.	N/A	Tan, Tan, Barkham, Yan, & Zhu^53^
China, Shenyang	5,696 undergraduates aged 17 to 25 years	Cross-sectional, self-report questionnaire	51.3% of undergraduates	Clinical diagnosis by a dermatologist	Significant risk factors: anxiety, depression, diet (fried food, high fat food and spicy food intake), dysmenorrhoea, family history (first- and second-degree relatives), insomnia (frequent), lack of sleep (<8 h/day), mental stress, menstrual disorder, sex, skin type (oily, mixed), study pressure Significant protective factors: computer usage (<2 h/day), diet (frequent fruit intake), skin type (dry, neutral) Insignificant factors: age	N/A	Pillsbury’s diagnostic criteria	Presence of any acne lesion	Wei, *et al*., 2010
France	2266 individuals aged 15–24 years	Cross-sectional study, self-report questionnaire	60.7% of the surveyed population	Self-reported acne	Significant risk factors: cannabis use, diet (chocolates/sweets intake) Significant protective factors: tobacco use Insignificant factors: alcohol intake, BMI, diet (carbonated drink, dairy, fast food intake)	N/A	N/A	Presence of and types of inflammatory and non-inflammatory acne lesions present determined via questionnaire responses	Wolkenstein, *et al*., 2015
Europe, 7 countries (Belgium, Czech Republic, Slovak Republic, France, Italy, Poland, Spain)	10,521 individuals aged 15–24 years	Cross-sectional, online self-report questionnaire	57.8% of individuals (adjusted); Lowest prevalence rate was 42.2% in Poland while the highest rate was 73.5% in Czech Republic and Slovak Republic	Self-reported acne	Significant risk factors: country of residence (Czech Republic, Slovak Republic), diet (chocolate intake), family history (parents) Significant protective factors: age, country of residence (Belgium and Poland), tobacco use Insignificant factors: alcohol intake, BMI, cannabis use, diet (carbonated drink, dairy, fruit juice, ice cream, milk, pasta/rice/semolina, sweets intake), sex	N/A	N/A	Self-report	Wolkenstein, *et al*., 2018
China, Guangdong	3,163 students aged 10–18 years	Cross-sectional, self-report questionnaire	53.5% of students	Clinical diagnosis	Significant risk factors: age, lack of sleep, skin type (oily, mixed, neutral), use of cosmetics Insignificant factors: diet (high fat food, seafood and sweets intake), regularity of menses	Significant risk factors associated with increased acne severity: age	Numbers and types of inflammatory and non-inflammatory acne lesions	Presence of any acne lesion	Wu, *et al*.^54^
China	1,555 volunteers and 4834 of the volunteers’ first-degree relatives	Cross-sectional, self-report questionnaire and telephone interviews	62.7% of volunteers and 27.1% of their first-degree relatives	Self-reported acne for both volunteers and their first-degree relatives; for those with uncertain acne status, acne status was confirmed via clinical diagnosis by a dermatologist	Significant risk factors: family history (first-degree relatives)	N/A	N/A	Acne in participants and their first-degree relatives was determined via participant report and telephone confirmation with first-degree relatives; dermatologist analysis for those with unclear acne status	Xu, *et al*.^55^
Singapore	94 secondary school students, mean age = 14.9 years	Cross-sectional, self report questionnaires	95% in male participants and 92% in female participants	Self-reported acne and clinical classification of acne severity by an observer	N/A	Significant risk factors associated with increased acne severity: sebum level, mental stress	Plewig & Kligman severity grading system	N/A	Yosipovitch, *et al*.^56^
**Longitudinal design**									
USA	4273 males aged 9–15 years when the study started	Longitudinal study (from 1996 to 1999), self-report questionnaires	N/A	Self-reported acne status	Significant risk factors: diet (skim milk intake) Insignificant factors: diet (calcium, chocolate, dairy other than milk, fat from dairy, French fries, low fat milk, pizza, total fat, total milk, total vitamin A, total vitamin D, types of fat, vitamin A from food, vitamin D from food, whole milk intake)	N/A	N/A	Responses to the question “Compared to other people your age, how would you describe your acne?”	Adebamowo, *et al*., 2008
Norway, Oslo	2489 students who were 15–16 years when the study started	Longitudinal study for 3 years, self-report questionnaires	13.9% of students had moderate to severe acne (general acne prevalence was not reported)	Self-reported acne status	N/A	Significant risk factors associated with increased acne severity: diet (high dairy intake in females, full-fat dairy intake in the study population as a whole) Insignificant factors: diet (semi-skimmed diary, skimmed dairy intake, moderate dairy intake in boys or the study population as a whole)	Responses to the question “‘In the last week, have you had pimples?”		Ulvestad, Bjertness, Dalgard & Halvorsen, 2017
Case-control design									
Afghanistan, Kabul	279 cases (defined as having moderate-severe acne) and 279 controls aged 10 to 24	Case-control study, self-report questionnaire	N/A	Clinical diagnosis by a dermatologist	N/A	Significant risk factors associated with increased acne severity: diet (chocolate, egg, low fat milk, potato chips, whole milk intake), family history (siblings) Significant protective factors: diet (chicken intake), dieting, physical exercise Insignificant factors: age of menarche, cannabis use, diet (vegetables intake)	Global Acne Severity Scale	N/A	Aalemi, Anwar, & Chen, 2019
Italy, 15 cities in Italy	205 cases from a dermatology clinic and 358 controls aged 10–24 years	Case-control study, interview using standardised questions	N/A	Clinical diagnosis by a dermatologist	N/A	Significant risk factors associated with increased acne severity: BMI (above 18.5), diet (milk intake), family history (first degree relatives), Significant risk factors associated with decreased acne severity: BMI (low), diet (fish intake) Insignificant factors: diet (bread/pasta, cheese/yoghurt, cured meat, desserts, fruits/vegetables, milk-free chocolate, red meat intake), menstrual characteristics, smoking status, use of contraceptives (oral)	Global score based on the numbers and types of inflammatory and non-inflammatory acne lesions	Cases were diagnosed with moderate to severe acne at a dermatology department; controls had no or mild acne lesions who did not receive acne treatment	Di Landro, *et al*., 2012
Italy, 12 cities in Italy	248 female cases and 270 controls aged 25 years and above	Case-control study, self-report questionnaire	N/A	Clinical diagnosis by a dermatologist	Significant risk factors (in adult females): diet (fish, fruit, vegetable intake), family history (first-degree relatives), hirsutism, job (office worker), mental stress, pregnancy (never), onset of acne in adolescence Insignificant factors: alcohol intake, diet (beef, cakes/sweets, chocolates, dairy and high-starch foods intake) education level, smoking status, regularity of menses, use of contraceptives (oral)	N/A	N/A	Global score based on the numbers and types of inflammatory and non-inflammatory acne lesions	Di Landro, *et al*., 2016
UK, Leeds	204 cases aged and 1203 of their first-degree relatives and 144 controls and their 856 first-degree relatives, all individuals were aged 25 and older	Case-control study	N/A	For cases: clinical diagnosis of acne vulgaris by a dermatologist For relatives of both cases and controls: self-reported acne status	Significant risk factors: family history (first-degree relatives) Insignificant factors: use of contraceptives	N/A	N/A	Cases: clinical acne diagnosis, criteria not specified Relatives: self-report	Goulden, McGeown, & Cunliffe, 1999
Egypt, Benha	100 cases and 100 controls	Case-control study, self-report questionnaire	N/A	Clinical diagnosis of acne vulgaris by a dermatologist	Significant risk factors: diet, family history, mental stress, smoking status, sun exposure	N/A	N/A	Global Acne Grading system	Ibrahim, Salem, El‐Shimi, Baghdady & Hussein^57^
Malaysia, Kuala Lumpur	88 individuals aged 18 to 30 years	Case-control study, self-report questionnaire	N/A	Clinical diagnosis by a dermatologist	Significant risk factors: diet (high glycaemic load, ice cream, milk intake), family history (parents, siblings) Insignificant factors: BMI, body fat percentage, diet (carbohydrate, chocolate, cheese, energy, fat, fibre, nut, protein, selenium, vitamin A, vitamin E, yoghurt and zinc intake), height, weight	N/A	N/A	Controls scored 0 or 1 on the comprehensive acne severity scale; cases were receiving acne treatment at a dermatology clinic	Ismail, Manaf, & Azizan, 2012
Turkey, 7 different cities	3837 cases and 759 controls (median age = 20.4)	Case-control study, self-report questionnaire	N/A	Clinical diagnosis by dermatologists	N/A	Significant risk factors associated with increased acne severity: BMI, diet (chocolate, fruit juice intake), family history, living environment, sex, smoking status, Significant risk factors associated with decreased acne severity: diet (cookie, watermelon, white rice, whole grain bread intake) Insignificant factors: diet (intake of other studied foods)	Global score based on the numbers and types of inflammatory and non-inflammatory acne lesions	N/A	Karadağ, *et al*., 2019
China, Shanghai and Ningbo	364 cases and 295 controls aged 10 to 25	Case-control study	N/A	Clinical diagnosis by a dermatologist	Significant risk factors: BMI, family history of diabetes mellitus, family history of hypertension, family history of obesity	Significant risk factors associated with increased acne severity: BMI	Pillsbury grading scale	Presence of any acne lesion	Lu, *et al*., 2017
Italy, Lazio	93 cases (median age = 17) and 200 controls (median age = 16)	Case-control study, self-report questionnaire	N/A	Clinical diagnosis by a dermatologist	Significant risk factors: family history of diabetes, family history of hypercholestrolemia, family history of hypertension Significant protective factors: Mediterranean diet (diet with high consumption of fish, fruits, grains, legumes, nuts, olive oil, vegetables; low consumption of red meat; moderate consumption of alcohol, dairy and milk)	N/A	N/A	Cases were diagnosed with acne at the dermatology department of a hospital, criteria for choice of controls not stated	Skroza, *et al*.^58^
Malaysia, Georgetown	57 cases and 57 controls aged 14 and above	Case-control study, self-report questionnaire	N/A	Clinical assessment	Significant risk factors: diet (chocolate, milk intake), family history Insignificant factors: diet (carbonated drink, ice cream, nuts, potato chips, sweets and yoghurt intake), smoking status	N/A	N/A	Comprehensive Acne Severity Scale	Suppiah, *et al*.^59^
China, Shanghai	1037 cases and 1046 controls	Case-control study, self-report questionnaire	N/A	Clinical assessment by a dermatologist	Significant risk factors: anaemia, diet (fatty food, seafood, sugary food intake), family history, hypertrichosis, menstrual disorder, psychological disorder, skin type (oily, mixed), sleep duration	Significant risk factors associated with increased acne severity: family history, psychological disorder	Global Acne Grading System	Presence of any acne lesion	Wang, *et al*., 2016

### Demographic factors that influence acne presentation

Many papers have demonstrated that acne presentation is influenced by demographic factors. The onset of acne typically correlates with the onset of puberty, when sebum production increases^19^. As such, the prevalence of acne increases with increasing age, showing highest incidence in teenagers and a relatively low incidence in pre-pubertal children^19^. After reaching the late teenage years or young adulthood, acne prevalence rates follow a decreasing trend with increasing age^19,20^. The results of the reviewed articles generally followed this trend, with higher odds of acne in teenagers compared to young adults and children (Tables 2, 3).

**Table 2:** List of risk factors for acne presentation analysed in the articles and results obtained for each risk factor.

Factor	Studies showing		
	Significant Risk Factor for Acne	Significant Protective Factor for Acne	Insignificant factor
**Demographic**			
Age (increasing)	Aksu, *et al*., 2012; Hogewoning, *et al*., 2009; Karciauskiene, Valiukeviciene, Gollnick & Stang, 2014; Park, Kwon, Min, Yoon, & Suh, 2015; Schäfer, Nienhaus, Vieluf, Berger, & Ring, 2001; Wu, *et al*.^54^;	Ali, *et al*.^50^ (younger age); Wolkenstein, *et al*., 2018	Wei, *et al*., 2010
Computer usage	N/A	Wei, *et al*., 2010 (less than 2 h/day)	N/A
Job (Office worker)	**Di Landro**, ***et al*., 2016**	N/A	N/A
Marital status	N/A	Ali, *et al*.^50^ (married)	N/A
Parent’s educational level	N/A	N/A	Bagatin, *et al*., 2014
Sex	Hogewoning, *et al*., 2009 (female); Rombouts, Nijsten & Lambert, 2006 (males); Schäfer, Nienhaus, Vieluf, Berger, & Ring, 2001 (male); Wei, *et al*., 2010 (male);	Ali, *et al*.^50^ (male)	Aksu, *et al*., 2012; Park, Kwon, Min, Yoon, & Suh, 2015; Wolkenstein, *et al*., 2018
Socioeconomic status	N/A	N/A	Schäfer, Nienhaus, Vieluf, Berger, & Ring, 2001;
Years of education	N/A	N/A	**Di Landro**, ***et al*., 2016** (personal educational level); Duquia, *et al*., 2017
**Genetic and/or Hormonal**			
Family History (parents with acne)	Al Hussein, *et al*., 2016; Karciauskiene, Valiukeviciene, Gollnick & Stang, 2014; **Ismail, Manaf, & Azizan, 2012** (close relatives, eg: parents and siblings); Wolkenstein, *et al*., 2018	N/A	N/A
Family History (first-degree relatives with acne)	**Di Landro**, ***et al*., 2016; Goulden, McGeown, & Cunliffe, 1999; Ibrahim, Salem, El-Shimi, Baghdady & Hussein**^57^ (family members included not specified); **Suppiah**, ***et al***.^59^ (family members included not specified); **Wang**, ***et al*., 2016** (family members included not specified); Wei, *et al*., 2010 (first- and second-degree relatives); Xu, *et al*.^55^;	N/A	Bagatin, *et al*., 2014
Height	N/A	Duquia, *et al*., 2017 (short height)	**Ismail, Manaf, & Azizan, 2012**
Onset of puberty	Karciauskiene, Valiukeviciene, Gollnick & Stang, 2014;	Ali, *et al*.^50^ (pre-menstrual stage)	Rombouts, Nijsten & Lambert, 2006
Pregnancy (never been pregnant)	**Di Landro**, ***et al****.***, 2016**	N/A	N/A
Personal history of acne in adolescence	**Di Landro**, ***et al****.***, 2016**	N/A	N/A
Regularity of menses	N/A	N/A	**Di Landro**, ***et al*., 2016**; Rombouts, Nijsten & Lambert, 2006; Wu, *et al*.^54^
Skin colour	N/A	Duquia, *et al*., 2017 (light skin phenotype)	Bagatin, *et al*., 2014
Skin type	**Wang**, ***et al*., 2016** (oily, mixed); Wei, *et al*., 2010 (oily/mixed); Wu, *et al*.^54^ (oily/mixed/neutral);	Ali, *et al*.^50^ (dry, normal, oily); Wei, *et al*., 2010 (dry/neutral);	N/A
Use of contraceptives (oral)	N/A	N/A	**Di Landro**, ***et al****.***, 2016;** **Goulden, McGeown, & Cunliffe, 1999**
Weight	N/A	N/A	**Ismail, Manaf, & Azizan, 2012**
**Medical History**			
Anaemia	**Wang**, ***et al****.***, 2016;**	N/A	N/A
Anxiety	Wei, *et al*., 2010	N/A	N/A
Depression	Wei, *et al*., 2010	N/A	N/A
Dysmenorrhoea	Wei, *et al*., 2010	N/A	N/A
Familial diabetes	**Lu**, ***et al****.***, 2017;** **Skroza**, ***et al****.*^58^**;**	N/A	N/A
Familial hypercholesterolemia	**Skroza**, ***et al****.*^58^	N/A	N/A
Familial hypertension	**Lu**, ***et al****.***, 2017;** **Skroza**, ***et al****.*^58^**;**	N/A	N/A
Family history of obesity	**Lu**, ***et al****.***, 2017;**	N/A	N/A
Hirsutism	**Di Landro**, ***et al****.***, 2016**	N/A	N/A
Hypertrichosis	**Wang**, ***et al****.***, 2016;**	N/A	N/A
Menstrual Disorder	**Wang**, ***et al****.***, 2016**; Wei, *et al*., 2010	N/A	N/A
Psychological disorder	Wang, *et al*., 2016;	N/A	N/A
**Diet**			
Diet (general)	**Ibrahim, Salem, El-Shimi, Baghdady & Hussein**^57^	N/A	**Ismail, Manaf, & Azizan, 2012** (energy intake);
Carbonated drink intake	Al Hussein, *et al*., 2016;	N/A	Karciauskiene, Valiukeviciene, Gollnick & Stang, 2014 (lemonade); **Suppiah**, ***et al****.*^59^; Wolkenstein, *et al*., 2015; Wolkenstein, *et al*., 2018
Dairy intake	*Adebamowo, et al., 2008* (skim milk only); **Ismail, Manaf, & Azizan, 2012** (frequent intake of milk and ice cream); **Suppiah**, ***et al****.*^59^ (milk);	N/A	*Adebamowo, et al., 2008* (dairy fat, dairy other than milk, total milk, whole milk, low-fat milk); Al Hussein, *et al*., 2016 (milk, cheese, yoghurt); **Di Landro**, ***et al****.***, 2016**; Duquia, *et al*., 2017 (whole milk, low fat milk, cheese, yoghurt); **Ismail, Manaf, & Azizan, 2012** (cheese and yoghurt); Karciauskiene, Valiukeviciene, Gollnick & Stang, 2014; **Suppiah**, ***et al***.^59^ (ice cream, yoghurt); Wolkenstein, *et al*., 2015; Wolkenstein, *et al*., 2018 (dairy in general, milk, type of milk and ice cream);
Fast food intake	Aksu, *et al*., 2012 (high intake of sausages and cakes); Wei, *et al*., 2010;	N/A	*Adebamowo, et al., 2008* (French fries and pizza); Karciauskiene, Valiukeviciene, Gollnick & Stang, 2014 (hamburgers and pizza); Park, Kwon, Min, Yoon, & Suh, 2015 (pizza); Wolkenstein, *et al*., 2015;
Fat intake	Aksu, *et al*., 2012; Al Hussein, *et al*., 2016; **Wang**, ***et al*., 2016** (fatty food); Wei, *et al*., 2010	N/A	*Adebamowo, et al., 2008* (total fat, types of fat); **Ismail, Manaf, & Azizan, 2012;** **Suppiah**, ***et al****.*^59^ (potato chips); Wu, *et al*.^54^;
Fish intake	**Di Landro**, ***et al****.***, 2016** (low intake of fish); **Wang**, ***et al****.***, 2016** (seafood);	Al Hussein, *et al*., 2016;	Karciauskiene, Valiukeviciene, Gollnick & Stang, 2014; Wu, *et al*.^54^ (seafood);
Fruits and vegetables intake	**Di Landro**, ***et al****.***, 2016** (low intake of fruits and vegetables);	Aksu, *et al*., 2012; Al Hussein, *et al*., 2016; Wei, *et al*., 2010 (frequent fruit intake only, not vegetables);	**Ismail, Manaf, & Azizan, 2012** (fibre); Karciauskiene, Valiukeviciene, Gollnick & Stang, 2014; Wolkenstein, *et al*., 2018 (fruit juice);
Glycaemic load	**Ismail, Manaf, & Azizan, 2012 (high)**	N/A	N/A
Intake of rice/pasta/ semolina	N/A	N/A	Wolkenstein, *et al*., 2018
Irregular meals	N/A	N/A	Al Hussein, *et al*., 2016
Lack of nutritional information	N/A	N/A	Al Hussein, *et al*., 2016
Meat intake	N/A	N/A	**Di Landro**, ***et al****.***, 2016** (beef); **Ismail, Manaf, & Azizan, 2012** (protein); Karciauskiene, Valiukeviciene, Gollnick & Stang, 2014; Park, Kwon, Min, Yoon, & Suh, 2015;
Mediterranean diet (high intake of legumes, grains, fish, olive oil, fruits, vegetables and nuts; low intake of meat; moderate intake of milk, dairy products and alcohol)	N/A	**Skroza**, ***et al****.*^58^	N/A
Nut intake	N/A	N.A	**Ismail, Manaf, & Azizan, 2012;** **Suppiah**, ***et al***.^59^;
Spicy food intake	Wei, *et al*., 2010	Ali, *et al*.^50^ (intake of non-spicy food)	N/A
Sugar/chocolates intake	Aksu, *et al*., 2012 (high sugar intake, high intake of pastries and cakes); Al Hussein, *et al*., 2016 (sweets); Park, Kwon, Min, Yoon, & Suh, 2015; **Suppiah**, ***et al****.*^59^ (chocolate); **Wang**, ***et al****.***, 2016**; Wolkenstein, *et al*., 2015; Wolkenstein, *et al*., 2018 (chocolate)	N/A	*Adebamowo, et al., 2008*; **Di Landro**, ***et al****.***, 2016** (cakes, chocolate, sweets, high-starch foods); Duquia, *et al*., 2017; **Ismail, Manaf, & Azizan, 2012** (chocolate, carbohydrates); Karciauskiene, Valiukeviciene, Gollnick & Stang, 2014 (sweets); **Suppiah**, ***et al****.*^59^ (sweets); Wolkenstein, *et al*., 2018 (sweets); Wu, *et al*.^54^ (sweet food);
Vitamin and mineral intake	N/A	N/A	*Adebamowo, et al., 2008* (vitamin A, vitamin D and calcium); **Ismail, Manaf, & Azizan, 2012** (vitamin A, vitamin E, zinc, selenium); Rombouts, Nijsten & Lambert, 2006 (multivitamin)
White bread intake	Al Hussein, *et al*., 2016;	N/A	N/A
BMI (overweight/obese)	Aksu, *et al*., 2012; Al Hussein, *et al*., 2016; Hogewoning, *et al*., 2009; Karciauskiene, Valiukeviciene, Gollnick & Stang, 2014; **Lu**, ***et al****.***, 2017**; Park, Kwon, Min, Yoon, & Suh, 2015;	N/A	Duquia, *et al*., 2017; **Ismail, Manaf, & Azizan, 2012** (as well as body fat percentage); Wolkenstein, *et al*., 2015; Wolkenstein, *et al*., 2018
**Substance Use**			
Alcohol intake	N/A	N/A	**Di Landro**, ***et al****.***, 2016**; Karciauskiene, Valiukeviciene, Gollnick & Stang, 2014; Schäfer, Nienhaus, Vieluf, Berger, & Ring, 2001; Wolkenstein, *et al*., 2015; Wolkenstein, *et al*., 2018;
Cannabis use	Wolkenstein, *et al*., 2015;	N/A	Wolkenstein, *et al*., 2018
Drug usage	N/A	N/A	Rombouts, Nijsten & Lambert, 2006
Number of cigarettes smoked/day	Schäfer, Nienhaus, Vieluf, Berger, & Ring, 2001 (dose-dependent relationship);	Rombouts, Nijsten & Lambert, 2006 (females)	Rombouts, Nijsten & Lambert, 2006 (males)
Smoking duration	N/A	Rombouts, Nijsten & Lambert, 2006 (females)	Rombouts, Nijsten & Lambert, 2006 (males)
Smoking status (cigarettes)	Al Hussein, *et al*., 2016; **Ibrahim, Salem, El-Shimi, Baghdady & Hussein**^57^; Schäfer, Nienhaus, Vieluf, Berger, & Ring, 2001;	Rombouts, Nijsten & Lambert, 2006 (females)	**Di Landro**, ***et al****.***, 2016**; Duquia, *et al*., 2017; Karciauskiene, Valiukeviciene, Gollnick & Stang, 2014; Rombouts, Nijsten & Lambert, 2006 (males); **Suppiah**, ***et al****.*^59^
Tobacco use	N/A	Wolkenstein, *et al*., 2015; Wolkenstein, *et al*., 2018;	N/A
**Living Environment, Stress and Emotional factors**			
Country of residence	Wolkenstein, *et al*., 2018 (living in Czech Republic or Slovak Republic)	Wolkenstein, *et al*., 2018 (living in Poland or Belgium)	N/A
Insomnia	Wei, *et al*., 2010 (frequent)	N/A	N/A
Living environment (Urban/rural)	Hogewoning, *et al*., 2009 (urban);	Aksu, *et al*., 2012 (urban)	Al Hussein, *et al*., 2016;
Mental stress	**Di Landro**, ***et al****.***, 2016;** **Ibrahim, Salem, El-Shimi, Baghdady & Hussein**^57^; Wei, *et al*., 2010;	N/A	N/A
Physical exercise	N/A	N/A	Rombouts, Nijsten & Lambert, 2006
Sleep duration (lack of sleep)	**Wang**, ***et al****.***, 2016** (less than 8 h); Wei, *et al*., 2010 (less than 8 h); Wu, *et al*.^54^;	N/A	Park, Kwon, Min, Yoon, & Suh, 2015 (less than 9 h);
Study pressure	Wei, *et al*., 2010	N/A	N/A
Sun exposure	**Ibrahim, Salem, El-Shimi, Baghdady & Hussein**^57^	N/A	N/A
Sunbed usage	N/A	N/A	Rombouts, Nijsten & Lambert, 2006
**Skincare**			
Frequency of face washing/day	N/A	Aksu, *et al*., 2012 (with tap water)	Park, Kwon, Min, Yoon, & Suh, 2015 (with cleanser);
Use of moisturisers/ cosmetics	Wu, *et al*.^54^;	N/A	Park, Kwon, Min, Yoon, & Suh, 2015;

Study design is indicated via text colour. Black text indicates a cross-sectional design, *italic* text indicates a longitudinal design and bold text indicates a case-control design.

**Table 3:** Strength of association of risk factors with acne presentation.

Study	Sample size	Odds Ratio (OR) or Prevalence Ratio (PR)	Odds Ratio (OR) or Prevalence Ratio (PR) 95% CI	p-value	References
**Demographics**					
**Age**					
Aksu, *et al*., 2012	2300	OR 2.38 (age 15–16) OR 1.59 (age 17–18)	OR 1.95–2.92 (age 15–16) OR 1.26–2.01 (age 17–18)	<0.05 (significant)	17–18 year olds or 15–16 year olds, respectively, with ref. to 13–14 year olds, OR adjusted for gender and living environment
Ali, *et al*.^50^^†^	1000	OR 3.52	95% CI not reported	0.013	Adults with ref to teenagers (specific age not specified)
Hogewoning, *et al*., 2009	1394	OR 3.3 (age 13 and 14) OR 2.6 (age 15 and 16)	OR 1.8–5.9 (age 13 and 14) OR 0.87–7.5 (age 15 and 16)	<0.05 (significant)	Individuals in urban schools aged 13–14 or 15–16, respectively, with ref to individuals in urban schools aged 9–12, adjusted for sex, BMI, type of school
Karciauskiene, Valiukeviciene, Gollnick & Stang, 2014	1229	OR not reported	OR not reported	N/A	Acne prevalence increased with age
Park, Kwon, Min, Yoon, & Suh, 2015	693	PR 1.99 (calculated)	PR Not reported	<0.001	Percentage of students in the upper grades with acne, with ref. to percentage of students in the lower grades with acne
Schäfer, Nienhaus, Vieluf, Berger, & Ring, 2001^†^	896	OR 1.49	OR 1.35–1.64	<0.001	Comparison and ref groups not defined, OR adjusted p-value is for the linear trend where age was positively correlated with acne prevalence
Wolkenstein, *et al*., 2018	10521	OR 0.806 (18–20 years) OR 0.728 (21–24 years)	OR 0.700 to 0.928 (18–20 years) OR 0.639 to 0.830 (21–24 years)	<0.0001	Age (18–20 years or 21–24 years, respectively) with ref. to age 15–17 years
Wu, *et al*.^54^	3163	OR 1.23	OR 1.18–1.29	<0.001	Reference age group not reported; higher ages positively associated with acne prevalence
**Computer usage**					
Wei, *et al*., 2010	5696	OR 0.891	95% CI not reported	N/A	Computer usage of <2 h per day; ref group not stated
**Job**					
**Di Landro**, ***et al****.***, 2016**	**518**	**OR 2.24**	**OR 1.24–4.06**	**0.007**	**Those who work as office worker with ref. to those who are housewives or unemployed**
**Marital status**					
Ali, *et al*.^50^	1000	OR 0.158	95% CI not reported	0.016	Individuals who are married with ref. to individuals who are not married
**Sex**					
Ali, *et al*.^50^	1000	OR 0.255	95% CI not reported	0.04	Males with ref. to females
Hogewoning, *et al*., 2009^†^	1394	OR 0.313	OR 0.164–0.588	<0.05 (significant)	Males in urban schools with ref. to females in urban schools, adjusted for age, BMI and type of school
Rombouts, Nijsten & Lambert, 2006^†^	594	OR 2.27	OR 1.47–3.23	<0.001	Males with 20 or more retentional or inflammatory acne lesions with ref to females with 20 or more retentional or inflammatory acne lesions, OR adjusted for stage of puberty, BMI, type of education at secondary school level, use of oral contraceptives, smoking and alcohol consumption
Schäfer, Nienhaus, Vieluf, Berger, & Ring, 2001	896	OR 1.53	OR 1.09–2.14	<0.05 (significant)	Male with ref. to female, OR adjusted
Wei, *et al*., 2010	5696	OR 1.405	95% CI not reported	N/A	Male with ref. to female
**Genetic or Hormonal**					
**Family History**					
Al Hussein, *et al*., 2016	148	OR 4.784	OR 2.337–9.794	<0.001	Family history (parental acne) with ref. to no family history
**Di Landro**, ***et al****.***, 2016**	**518**	**OR 3.02 (parental acne)** **OR 2.40 (acne in siblings)**	**OR 1.80–5.06 (parental acne)** **OR 1.46–3.94 (acne in siblings)**	**<0.001 (parental acne)** **0.001 (sibling acne)**	**Family history (parental or sibling acne, respectively) with ref. to no family history (absence of parental or sibling acne, respectively), OR adjusted for age**
**Goulden, McGeown, & Cunliffe, 1999**	**348**	**OR 3.93**	**OR 2.79–5.51**	**<0.05 (significant)**	**Risk of developing acne in adulthood in first-degree relatives of acne cases with ref. to risk of developing acne in adulthood in first-degree relatives of controls**
**Ismail, Manaf, & Azizan, 2012**	**88**	**PR 2.40 (calculated)**	**N/A**	**<0.001**	**Chi squared test conducted; cases with a family history of acne with ref. to controls with a family history of acne**
Karciauskiene, Valiukeviciene, Gollnick & Stang, 2014	1229	OR 2.1 (maternal acne) OR 1.7 (paternal acne) OR 2.6 (both maternal and paternal acne)	OR 1.4–3 (maternal acne) OR 1.1–2.6 (paternal acne) OR 1.6–4.1 (both maternal and paternal acne)	N/A	Family history (maternal, paternal or both maternal and paternal acne, respectively) with ref. to no family history (absence of maternal, paternal or both maternal and paternal acne, respectively), OR adjusted for age
**Suppiah**, ***et al****.*^59^	**114**	**PR 3.05 (calculated)**	**N/A**	**<0.001**	**Acne cases with a family history of acne in immediate family members with ref. to controls with a family history of acne in immediate family members**
**Wang**, ***et al****.***, 2016**	**2083**	**PR 3.30 (calculated)**	**N/A**	**<0.001**	**Cases with a family history of acne with ref. to controls with a family history of acne**
Wei, *et al*., 2010	5696	OR 4.722	95% CI not reported	<0.001	Family history (family members included not specified) with ref. to no family history
Wolkenstein, *et al*., 2018	10521	OR 3.077 (maternal acne) OR 2.700 (paternal acne)	OR 2.743 to 3.451 (maternal acne) OR 2.391 to 3.049 (paternal acne)	<0.0001 for both maternal and paternal acne	Family history (maternal or paternal acne, respectively) with ref to no family history (absence of maternal or paternal acne, respectively)
Xu, *et al*.^55^	1555	OR 4.05	OR 3.45–4.76	<0.001	Risk of acne vulgaris in a relative of a individual with acne vulgaris with ref. to the risk of acne vulgaris in a relative of an individual with no acne vulgaris
**Height**					
Duquia, *et al*., 2017	2201	PR 1.06 (second tertile) PR 1.07 (third tertile)	PR 1.01–1.11 (second tertile) PR 1.02–1.13 (third tertile)	0.006	Third tertile of height or second tertile of height, respectively with ref. to first tertile of height, PR adjusted
**Onset of puberty**					
Ali, *et al*.^50^^†^	1000	OR 5.99	95% CI not reported	0.014	Females in the post-menstrual stage with ref. to females in the pre-menstrual stage
Karciauskiene, Valiukeviciene, Gollnick & Stang, 2014	1229	OR 3.1 (females) OR 4.9 (males)	OR 1.04–9.4 (females) OR 1.3–19 (males)	N/A	For females: girls with menses with ref. to girls without, OR adjusted for age For males: boys with facial hair growth with ref. to boys without, OR adjusted for age
**Pregnancy**					
**Di Landro**, *et al*.,** 2016**	**518**	**OR 1.71**	**OR 1.06–2.78**	**0.02**	**Having no previous pregnancies with ref. to having a previous pregnancy**
**Personal history of acne in adolescence**					
**Di Landro**, ***et al****.***, 2016**	**518**	**OR 5.44**	**OR 3.43–8.61**	**<0.001**	**Having a personal history of acne during adolescence with ref to no personal history of acne during adolescence**
**Skin Colour**					
**Duquia**, ***et al****.***, 2017**	**2201**	**PR 0.91**	**PR 0.86–0.96**	**<0.001**	**Light skin colour with ref. to dark skin colour, PR adjusted**
**Skin type**					
Ali, *et al*.^50^	1000	OR 0.164 (dry) OR 0.120 (normal) OR 0.132 (oily)	95% CI not reported	0.010	Skin type (dry, normal or oily, respectively) with ref. to semi oily skin type
**Wang**, ***et al****.***, 2016**	**2083**	**Not reported**	**Not reported**	**<0.001**	**Individuals with oily or mixed skin type were significantly more likely to have acne**
Wei, *et al*., 2010	5696	OR 1.110 (oily) OR 1.025 (mixed) OR 0.421 (dry) OR 0.422 (neutral)	95% CI not reported	N/A	Comparison and Reference groups not specified
Wu, *et al*.^54^	3163	OR 11.01 (oily) OR 14.26 (mixed) OR 1.69 (neutral)	OR 8.14–14.89 (oily) OR 10.22–19.89 (mixed) OR 1.32–2.16 (neutral)	<0.001 (oily, mixed and neutral)	Skin type (oily, mixed or neutral, respectively) with ref. to dry skin
**Medical History**					
**Anaemia**					
**Wang**, ***et al****.***, 2016**	**2083**	**Not reported**	**Not reported**	**<0.001**	**Those with anaemia were significantly more likely to have acne**
**Anxiety**					
Wei, *et al*., 2010	5696	OR 1.314	95% CI not reported	Not reported	Presence of clinical anxiety with ref. to absence of clinical anxiety
**Depression**					
Wei, *et al*., 2010	5696	OR 1.197	95% CI not reported	Not reported	Presence of clinical depression with ref. to absence of clinical depression
**Dysmenorrhoea**					
Wei, *et al*., 2010	5696	OR 1.339	95% CI not reported	Not reported	Presence of dysmenorrhoea with ref. to absence of dysmenorrhoea
**Familial diabetes**					
**Lu**, ***et al****.***, 2017**	**659**	**OR 2.697**	**OR 1.565–4.647**	**<0.001**	**Individuals with a family history of diabetes with ref. to Individuals without a family history of diabetes**
**Skroza**, ***et al****.*^58^	**293**	**OR 3.32**	**OR 1.27–8.63**	**<0.001**	**Individuals with familial diabetes with ref. to Individuals without familial diabetes, OR adjusted using a backward elimination model**
**Familial hypercholesterolemia**					
**Skroza**, ***et al****.*^58^	**293**	**OR 8.79**	**OR 1.67–46.22**	**<0.001**	**Individuals with familial hypercholesterolemia with ref. to Individuals without familial hypercholesterolemia, OR adjusted using a backward elimination model**
**Familial hypertension**					
**Lu**, ***et al****.***, 2017**	**659**	**OR 3.511**	**OR 1.977–6.233**	**<0.001**	**Individuals with a family history of hypertension with ref. to Individuals without a family history of hypertension**
**Skroza**, ***et al****.*^58^	**293**	**OR 2.73**	**OR 1.07–6.96**	**<0.05 (significant)**	**Individuals with familial hypertension with ref. to Individuals without familial hypertension, OR adjusted using a backward elimination model**
**Family history of obesity**					
**Lu**, ***et al****.***, 2017**	**659**	**OR 1.844**	**OR 1.242–4.407**	**0.032**	**Individuals with a family history of obesity with ref. to Individuals without a family history of obesity**
**Hirsutism**					
**Di Landro**, ***et al****.***, 2016**	**518**	**OR 3.50**	**OR 1.42–8.60**	**0.006**	**Presence of hirsutism with ref. to absence of hirsutism**
**Hypertrichosis**					
**Wang**, ***et al****.***, 2016**	**2083**	**Not reported**	**Not reported**	**<0.001**	**Those with hypertrichosis were significantly more likely to have acne**
**Menstrual disorder**					
**Wang**, ***et al****.***, 2016**	**2083**	**Not reported**	**Not reported**	**<0.001**	**Those with menstrual disorder were significantly more likely to have acne**
Wei, *et al*., 2010	5696	OR 1.501	95% CI not reported	N/A	Presence of menstrual disorder with ref. to absence
**Psychological disorders**					
**Wang**, ***et al****.***, 2016**	**2083**	**Not reported**	**Not reported**	**<0.001**	**Those with acne were significantly more likely to have psychological disorders**
**Diet**					
**Carbonated drink intake**					
Al Hussein, *et al*., 2016	148	OR 7.427	OR 3.548–15.55	<0.0001	Consumption of >200 ml of carbonated beverages frequently or daily, with ref. to consumption of >200 ml of carbonated beverages infrequently
**Dairy intake**					
*Adebamowo, et al., 2008*	*4273*	*PR 1.19*	*PR 1.01–1.40 (skim milk)*	*0.02(skim milk)*	*Males who had ≥2 servings of skim milk per day with ref. to males who had ≤ 1 serving of skim milk per week, PR adjusted for age, onset of puberty, BMI, daily energy intake*
Duquia, *et al*., 2017	2201	PR 1.05	PR 1.00–1.11	0.05	Daily yoghurt consumption with ref. to no yoghurt consumed every day
**Ismail, Manaf, & Azizan, 2012**	**88**	**OR 3.99 (milk)** **OR 4.47 (ice cream)**	**OR 1.39–11.43 (milk)** **OR 2.44–19.72 (ice cream)**	**<0.008 (milk)** **<0.001 (ice cream)**	**Consumption of milk or ice cream, respectively, ≥ 1 time per week with ref. to <1 time per week**
**Suppiah**, ***et al****.*^59^	**114**	**OR 2.19**	**OR 1.04–4.65**	**0.039**	**Consumption of 2 or more glasses of milk per day with ref to seldom consuming milk**
**Fast food intake**					
Aksu, *et al*., 2012	2300	OR 1.24	OR 1.03–1.48	<0.05 (significant)	Eating sausages and burgers frequently with ref to infrequently
Wei, *et al*., 2010	5696	OR 1.174	95% CI not reported	N/A	Frequent consumption of fried food; ref group not stated
**Fat intake**					
Aksu, *et al*., 2012	2300	OR 1.39	OR 1.06–1.82	<0.05 (significant)	Unhealthy fat intake with ref. to healthy fat intake (avoid fried food, trying to keep their total fat consumption low and choosing low-fat chips); OR adjusted for age, gender and living environment
Al Hussein, *et al*., 2016	148	OR 6.919	OR 3.187–15.02	<0.0001	>100 g of dietary fat consumed 2–4 times per week with ref. to>100 g of dietary fat consumed less than 2 times per week
**Wang**, ***et al****.***, 2016**	**2083**	**Not reported**	**Not reported**	**<0.001**	**Having fatty food was significantly associated with acne occurrence**
Wei, *et al*., 2010	5696	OR 1.439	95% CI not reported	N/A	High fat diet; ref group not stated
**Fish intake**					
Al Hussein, *et al*., 2016	148	OR 0.126	OR 0.055–0.290	<0.0001	150 g of fish 2–4 times per week, with ref to. 150 g of fish less than 2 times per week
**Di Landro**, ***et al****.***, 2016**^†^	**518**	**OR 0.362**	**OR 0.172–0.763**	**0.008**	**Eating fish>3 days per week with ref to ≤3 days per week, OR adjusted for age**
**Wang**, ***et al****.***, 2016**	**2083**	**Not reported**	**Not reported**	**<0.001**	**Having seafood was significantly associated with acne occurrence**
**Fruits and vegetables intake**					
Aksu, *et al*., 2012	2300	Not reported	Not reported	0.026 (chi squared test)	≥5 servings of fruits and vegetables per day with ref. to <5 servings of fruits and vegetables per day
Al Hussein, *et al*., 2016	148	OR 0.205	OR 0.101–0.415	<0.0001	250 g of fruits and vegetables at least 2–3 times per day, with ref to 250 g of fruits and vegetables less than 2 times per day
**Di Landro**, *et al*., **2016**^**†**^	**518**	**OR 0.429**	**OR 0.221–0.833**	**0.01**	**Eating vegetables>3 days per week with ref to ≤3 days per week, OR adjusted for age**
Wei, *et al*., 2010	5696	OR 0.865	95% CI not reported	N/A	Frequent fruit consumption; ref group not stated
**Glycaemic Load**					
**Ismail, Manaf, & Azizan, 2012**	**88**	**OR 24.96**	**OR 2.285–272.722**	**<0.01**	**Glycaemic load of ≥175 with ref. to glycaemic load <175; OR adjusted for family history, education level, frequency of milk and ice cream intake**
**Mediterranean diet**					
**Skroza**, *et al*.^58^	**293**	**OR 0.31**	**OR 0.11–0.89**	**0.002**	**Mediterranean diet score ≥6 with ref. to Mediterranean diet score <6; OR adjusted using a backward elimination model**
**Spicy food intake**					
Ali, *et al*.^50^^†^	1000	OR 25.0	95% CI not reported	0.014	Individuals who consumed spicy food with ref. to individuals who consumed normal food
Wei, *et al*., 2010	5696	OR 1.146	95% CI not reported	N/A	Frequent consumption of spicy food; ref group not stated
**Sugars/chocolates intake**					
Aksu, *et al*., 2012	2300	OR 1.20 (pastries and cakes) OR 1.30 (sugars)	OR 1.01–1.43 (pastries and cakes) OR 1.05–1.60 (sugars)	<0.05 (significant)	Eating pastries and cakes frequently with ref to infrequently Unhealthy sugar intake with ref. to healthy sugar intake (avoid eating dessert, keeping total sugar intake low, rarely eating sweet treats between meals)
Al Hussein, *et al*., 2016	148	OR 5.938	OR 2.841–12.41	<0.0001	>100 g of sweets per day with ref. to <100 g of sweets per day
Park, Kwon, Min, Yoon, & Suh, 2015	693	OR 1.6	OR 1.13–2.24	<0.05 (significant)	Chocolates/sweets reported as subjects’ favourite food, with ref. to chocolates/sweets not reported as subjects’ favourite food, OR adjusted for age
**Suppiah**, ***et al****.*^59^	**114**	**OR 2.40**	**OR 1.08–5.33**	**0.030**	**Eating chocolate often with ref. to seldom eating chocolate**
**Wang**, ***et al****.***, 2016**	**2083**	**Not reported**	**Not reported**	**<0.001**	**Having sugary food was significantly associated with acne occurrence**
Wolkenstein, *et al*., 2015	2266	OR 1.99 (chocolate) OR 1.78 (sweets) OR 2.38 (chocolates and sweets) OR are all from multivariate analysis	OR 1.30–3.03 (chocolate) OR 1.04–3.06 (sweets) OR 1.31–4.31 (chocolates and sweets) OR are all from multivariate analysis	0.0004 (univariate p-value adjusted for age) multivariate p-value not presented	Consuming sweets, chocolates or both chocolates and sweets, respectively daily with ref. to consuming no sweets, no chocolates or both no chocolate and no sweets, respectively
Wolkenstein, *et al*., 2018	10521	OR 1.302 (Quartile 2) OR 1.286 (Quartile 3) OR 1.276 (Quartile 4)	OR 1.117 to 1.518 (Quartile 2) OR 1.108 to 1.493 (Quartile 3) OR 1.094 to 1.488 (Quartile 4)	0.0017	Quartile 2, 3 or 4 of chocolate consumption, respectively, with ref. to quartile 1 of chocolate consumption
**White bread intake**					
Al Hussein, *et al*., 2016	148	OR 4.259	OR 1.821–9.962	0.0007	>350 g per day with ref. to <350 g per day
**BMI**					
Aksu, *et al*., 2012	2300	OR 2.04 (normal) OR 2.56 (overweight)	OR 1.54–2.69 (normal) OR 1.55–4.24 (overweight)	<0.05 (significant)	Normal or overweight BMI, respectively with ref. to underweight BMI, OR adjusted for age, gender and living environment
Al Hussein, *et al*., 2016	148	OR 4.326	OR 1.492–12.54	0.004	BMI ≥ 25 (overweight and obese) with ref. to BMI < 25
Hogewoning, *et al*., 2009	1394	OR 0.68 (BMI < 17) OR 2.0 (BMI > 25)	OR 0.19–2.4 (BMI < 17) OR 0.93–4.3 (BMI > 25)	<0.05 (significant)	BMI > 25 at 18 years of age or BMI < 17 at 18 years of age, respectively, with ref to 25 ≥ BMI ≥ 17 at 18 years of age, OR adjusted for age and sex, type of school
Karciauskiene, Valiukeviciene, Gollnick & Stang, 2014	1229	OR 2.6	OR 1.6–4.3	N/A	BMI ≥ 25 at 18 years of age with ref to BMI < 25 at 18 years of age, OR adjusted for age and sex
**Lu**, ***et al****.***, 2017**	**659**	**OR 1.989**	**OR 1.148–3.445**	**0.013**	**Overweight/obese BMI with ref. to underweight/normal BMI**
Park, Kwon, Min, Yoon, & Suh, 2015	693	OR 2.7	OR 1.81–3.92	<0.05 (significant)	BMI ≥ 25 at 18 years old with ref. to BMI < 25 at 18 years old, OR adjusted for age and sex
**Substance Use**					
**Cannabis Use**					
Wolkenstein, *et al*., 2015	2266	Multivariate OR 2.88	Multivariate OR 1.55–5.37	0.0506 (univariate p-value adjusted for age) multivariate p-value not presented	Individuals who use cannabis on a regular basis with ref. to individuals who do not use cannabis
**Smoking Cigarettes**					
Al Hussein, *et al*., 2016	148	OR 2.859	OR 1.467–5.576	0.002	Smokers with ref. to non-smokers
Rombouts, Nijsten & Lambert, 2006	594	OR 0.41	OR 0.13–0.82	0.007	Females with acne smoked (defined as smoking ≥3 cigarettes per day for > 6 months) more often with ref. to females without acne, OR adjusted for BMI, acne treatment status and usage of oral contraceptives
Schäfer, Nienhaus, Vieluf, Berger, & Ring, 2001	896	OR 2.24 (ref to ex-smokers) OR 2.04 (ref to non-smokers)	OR 1.44–3.50 (ref to ex-smokers) OR 1.40–2.99 (ref to non-smokers)	<0.05 (significant)	Active smokers with ref. to ex-smokers or non-smokers, respectively, OR adjusted
Schäfer, Nienhaus, Vieluf, Berger, & Ring, 2001	896	Not reported	Not reported	<0.0001	Dose-dependent relationship between the number of cigarettes smoked per day and the prevalence of acne
**Tobacco use**					
Wolkenstein, *et al*., 2018	10521	OR 0.705 (current smoker) OR 0.910 (ex smoker)	OR 0.616 to 0.807 (current smoker) OR 0.780 to 1.062 (ex smoker)	<0.0001	Current tobacco smokers or ex tobacco smokers with ref. to individuals who have never smoked tobacco
Wolkenstein, *et al*., 2015	2266	Multivariate OR 0.44	Multivariate OR 0.30–0.66	0.0006 (univariate p-value adjusted for age) multivariate p-value not presented	Tobacco smokers who smoked>10 cigarettes per day with ref. to Tobacco non-smokers (0 cigarettes per day)
**Living Environment, Stress and Emotional factors**					
**Country of Residence**					
Wolkenstein, *et al*., 2018	10521	OR 0.456 (Poland) OR 1.963 (Czech and Slovak Republics) OR 0.780 (Belgium)	OR 0.384 to 0.540 (Poland) OR 1.620 to 2.379 (Czech and Slovak Republics) OR 0.638 to 0.953 (Belgium)	<0.0001 (overall p-value for country of residence)	Poland; Czech and Slovak Republics; and Belgium, respectively, with ref. to Spain
**Living environment**					
Aksu, *et al*., 2012	2300	OR 0.67	OR 0.56–0.79	<0.05 (significant)	Urban environment with ref. to semi-rural environment, OR adjusted for age and gender
Hogewoning, *et al*., 2009	1394	PR 64.5 (calculated)	N/A	<0.001	Percentage of urban school students with acne with ref. to percentage of rural school students with acne
**Mental Stress**					
**Di Landro**, ***et al****.***, 2016**	518	OR 2.95	OR 1.57–5.53	0.001	Very high self-reported stress during the last month with ref to mild self-reported stress in the last month
Wei, *et al*., 2010	5696	OR 1.557	95% CI not reported	Not reported	Comparison and Reference groups not specified
**Sleep duration (lack of sleep)**					
**Wang**, ***et al****.***, 2016**	**2083**	**Not reported**	**Not reported**	**<0.001**	**Those who got less than 8 h of sleep per night were significantly more likely to have acne**
Wei, *et al*., 2010	5696	OR 1.241	95% CI not reported	Not reported	Sleeping <8 h per night; reference group not indicated
Wei, *et al*., 2010	5696	OR 1.446	95% CI not reported	Not reported	Frequent insomnia; Comparison and Reference groups not specified
Wu, *et al*.^54^	3163	OR 1.23	OR 1.02–1.52	0.027	Individuals who were deprived of sleep with ref. to individuals who were not deprived of sleep
**Skincare**					
**Face washing**					
Aksu, *et al*., 2012	2300	OR 0.68	OR 0.48–0.99	<0.05 (significant)	Face washing ≥3 times per day with ref. to face washing 1 time per day, OR adjusted for age, gender and living environment
**Use of cosmetics**					
Wu, *et al*.^54^	3163	OR 1.58	OR 1.25–2.00	<0.001	Individuals who used makeup products on their face with ref. to individuals who did not use makeup products on their face

Study design is indicated via text colour. Black text indicates a cross-sectional design, *italic* text indicates a longitudinal design and bold text indicates a case-control design. ^†^Indicates that odds ratio and 95% CI has been converted so that the direction of the comparison and reference groups matches the other entries in the table.

**Figure 1:**
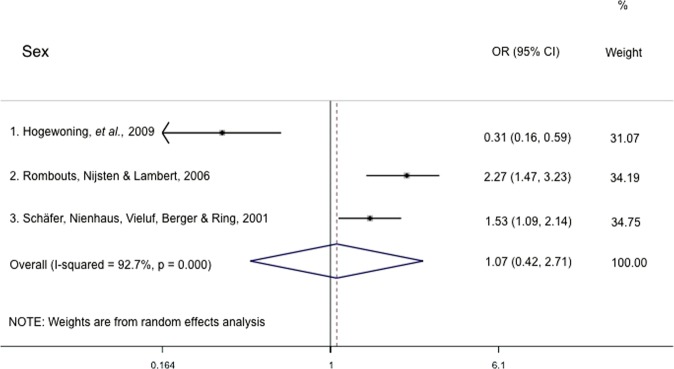
Individual and pooled odds ratio and 95% confidence intervals for acne presentation in association with male or female sex. Two studies were excluded from meta-analysis due to a lack of data, such as the odds ratio and/or 95% confidence interval.

Previous reviews have reported that the prevalence of acne is higher in females than males^20,21^. Similarly, the Global Burden of Disease Study conducted in 2010 estimated that the prevalence of acne was 8.96% in males, lower than the estimated prevalence of 9.81% in females^1^. Lynn *et al*.^21^ also noted higher acne prevalence in females at younger ages, possibly due to the earlier onset of puberty in females relative to males. However, the papers reviewed in this study showed mixed results, with only two papers revealing a higher odds of acne in females while another three demonstrated a higher odds of acne in males (Table 3). These results may be due to differences in the characteristics of the sampled population or country studied. Notably, in a study that found lower odds of acne in females than males, the odds ratio was adjusted for stage of puberty, removing the potential confounders of age and onset of puberty^22^. When the pooled odds ratio was calculated, an OR of 1.07 (95% CI 0.42–2.71; males with reference to females) was obtained (Fig. 1) suggesting that male sex is only associated with a slight increase in acne risk.

Relatively few studies considered other demographic factors. Factors including years of education, socioeconomic status and parent’s educational level did not significantly affect acne prevalence. Studies also found that low computer usage and marriage were protective for acne while working in an office was a risk factor for acne.

### Demographic factors that influence acne severity

Demographic factors also influence the severity of acne. The articles reviewed revealed that the odds of severe acne are higher in older teenagers compared to younger teenagers or preteens (Table 4). As sebum production increases during puberty, older teenagers tend to have higher sebum production compared to younger teenagers. High sebum levels favor the growth of *Propionibacterium acnes*, a species of bacterium implicated in inflammatory processes in acne and the development of inflammatory acne lesions which are typically associated with more severe acne^18,19^.

**Table 4:** Strength of association of risk factors with acne severity (moderate/severe acne).

Study	Sample size	Odds Ratio (OR) or Prevalence Ratio (PR)	Odds Ratio (OR) or Prevalence Ratio (PR) 95% CI	p-value	References
**Demographics**					
**Age**					
Aksu, *et al*., 2012	2300	Not reported	Not reported	0.000	Acne severity increased with age
Bagatin, *et al*., 2014	452	OR 17.413	OR 7.044–43.043	<0.001	17 years old with ref. to 10–11 years old
Ghodsi, Orawa, & Zouboulis^51^	1002	OR 2.2	OR 1.5–3.1	<0.0005	Age ≥ 17 years with ref. to age ≤ 16 years
Park, Kwon, Min, Yoon, & Suh, 2015	693	Not reported	Not reported	0.03	Higher mean acne severity score observed for students in upper grades compared to lower grades
Silverberg & Silverberg, 2014	9417	PR 7.12 (calculated)	Not reported	<0 0001	Prevalence of severe acne in children aged 17 with ref. to prevalence of severe acne in children aged 11
Wu, *et al*.^54^	3163	Not reported	Not reported	<0.001	Older ages are positively associated with more severe acne
**Number of children in the family**					
Silverberg & Silverberg, 2014	9417	Not reported	Not reported	0.02	Higher prevalence of severe acne in families with only one child at age 16–17
**Parent’s education level**					
Bagatin, *et al*., 2014	452	OR 1.726 (father) OR 1.973 (mother)	OR 1.151–2.588 (father) OR 1.317–2.958 (mother)	0.008 (father) 0.001 (mother)	Highest education level of parent (father or mother, respectively) being high school or below with ref. to highest education level of parent being college
Silverberg & Silverberg, 2014	9417	Not reported	Not reported	0.04	Higher prevalence of severe acne in families with a higher level of education at age 14–15
**Race**					
Silverberg & Silverberg, 2014	9417	Not reported	Not reported	0.0004	Higher prevalence of severe acne in Whites compared to other races at age 14–15
**Sex**					
Aksu, *et al*., 2012	2300	Not reported	Not reported	0.000	More severe acne was associated with being male
Kaminsky, Florez-White, Bagatin, & Arias, 2019	1384	PR 3.85 (calculated)	N/A	0.0001	Percentage of males with severe acne with ref. to percentage of females with severe acne
**Karadağ**, ***et al****.***, 2019**	**4596**	**Not reported**	**Not reported**	**<0.001**	**Mild acne occurred more frequently in females while severe acne occurred more frequently in males**
Silverberg & Silverberg, 2014	9417	Not reported	Not reported	0.02	Higher prevalence of severe acne in females than males at age 11–13
**Genetic or Hormonal**					
**Family History**					
**Aalemi, Anwar, & Chen, 2019;**	**558**	**OR 4.13**	**OR 2.55–6.69**	**<0.001**	**Sibling history of acne with ref. to no sibling history**
Bagatin, *et al*., 2014	452	OR 1.932	OR 1.261–2.961	0.002	Risk of non-comedonal acne in individuals with sibling with acne with ref. to individuals with siblings without acne
**Di Landro**, ***et al****.***, 2012**	**563**	**OR 3.41**	**OR 2.31–5.05**	**N/A**	**Family history (first-degree relatives) with ref. to no family history**
Ghodsi, Orawa, & Zouboulis^51^	1002	OR 1.7	OR 1.1–2.6	0.0017	Family history (parents and siblings) with ref. to no family history
**Karadağ**, ***et al****.***, 2019**	**4596**	**Not reported**	**Not reported**	**<0.001**	**Severe acne occurred more frequently in those with a family history of acne while mild acne occurred more frequently in those without a family history of acne**
Karciauskiene, Valiukeviciene, Gollnick & Stang, 2014	1229	OR 9.8 (maternal acne) OR 2.5 (paternal acne) OR 7.7 (maternal and paternal acne)	OR 2.9–33 (maternal acne) OR 0.9–6.3 (paternal acne) OR 2.1–27.9 (maternal and paternal acne)	N/A	Individuals with moderate/severe acne with family history (maternal, paternal or both maternal and paternal acne, respectively) with ref. to individuals with no acne with family history (absence of maternal, paternal or both maternal and paternal acne, respectively), OR adjusted for age
Karciauskiene, Valiukeviciene, Gollnick & Stang, 2014	1229	OR 2.7 (maternal acne) OR 2.3 (paternal acne) OR 4.2 (maternal and paternal acne)	OR 1.9–3.7 (maternal acne) OR 1.6–3.3 (paternal acne) OR 2.8–6.4 (maternal and paternal acne)	N/A	Individuals with mild acne with family history (maternal, paternal or both maternal and paternal acne, respectively) with ref. to individuals with no acne with family history (absence of maternal, paternal or both maternal and paternal acne, respectively), OR adjusted for age
**Menstrual chracteristics**					
Ghodsi, Orawa, & Zouboulis^51^	1002	Not reported	Not reported	0.015	Premenstrual phase was positively associated with acne severity
**Skin type**					
Aksu, *et al*., 2012	2300	Not reported	Not reported	0.000	More severe acne was associated with an oily skin type
Ghodsi, Orawa, & Zouboulis^51^	1002	OR 2.8	OR 1.7–4.5	<0.0005	Individuals with seborrhoeic skin with ref. to individuals with normal skin
Ghodsi, Orawa, & Zouboulis^51^	1002	OR 2.6	OR 1.6–4.2	<0.0005	Individuals who evaluated their skin type as oily skin with ref. to individuals who evaluated their skin type as normal skin
**Use of contraceptives (oral)**					
Rombouts, Nijsten & Lambert, 2006	594	PR 0.358 (calculated)	N/A	0.009	Percentage of females with moderate/severe acne who used of oral contraceptives with ref. to percentage of females without moderate/severe acne who used oral contraceptives
**Medical history**					
**Acanthosis Nigricans**					
Kaminsky, Florez-White, Bagatin, & Arias, 2019	1384	Not reported	Not reported	0.05 (significant)	Individuals who had Acanthosis Nigricans were more likely to have severe acne
**Acne characteristics**					
Aksu, *et al*., 2012	2300	Not reported	Not reported	0.000	Acne severity increased with the duration of acne
Kaminsky, Florez-White, Bagatin, & Arias, 2019	1384	Not reported	Not reported	0.015	Individuals who had acne in adolescence were more likely to have severe acne
**Hirsutism**					
Kaminsky, Florez-White, Bagatin, & Arias, 2019	1384	Not reported	Not reported	0.05 (significant)	Individuals with severe acne were more likely to have hirsutism
**Hyperseborrhea**					
Kaminsky, Florez-White, Bagatin, & Arias, 2019	1384	Not reported	Not reported	0.05 (significant)	Individuals with severe acne were more likely to have hyperseborrhea
**Gastrointestinal Conditions**					
Silverberg & Silverberg, 2014	9417	OR 3.09 (reflux) OR 2.14 (abdominal pain) OR 2.31 (nausea) OR 2.88 (food allergy)	OR 1.68–5.67 (reflux) OR 1.07–4.27 (abdominal pain) OR 1.51–3.53 (nausea) OR 1.28–6.47 (food allergy)	0 0003 (reflux) 0 03 (abdominal pain) 0 0001 (nausea) 0.01 (food allergy)	Presence of condition (reflux, abdominal pain, nausea and food allergy, respectively) with ref. to absence of condition
**Psychological disorders**					
Silverberg & Silverberg, 2014	9417	OR 2.46 (depression) OR 3.45 (anxiety) OR 2.09 (ADD/ADHD) OR 1.85 (insomnia)	OR 1.17–5.19 (depression) OR 2.16–5.50 (anxiety) OR 1.19–3.67 (ADD/ADHD) OR 1.09–3.11 (insomnia)	0.02 (depression and insomnia) <0.0001 (anxiety) 0.01 (ADD/ADHD)	Presence of condition (depression, anxiety, ADD/ADHD and insomnia, respectively) with ref. to absence of condition
**Sinopulmonary Disorders**					
Silverberg & Silverberg, 2014	9417	OR 2.35 (sinus infection) OR 2.01 (sore throat excluding strep throat) OR 2.38 (asthma) OR 2.64 (lung disease excluding asthma)	OR 1.48–3.73 (sinus infection) OR 1.38–2.94 (sore throat excluding strep throat) OR 1.08–5.25 (asthma) OR 1.08–6.46 (lung disease excluding asthma)	0 0003 (sinus infection, sore throat excluding strep throat) 0 03 (asthma and lung disease excluding asthma)	Presence of condition (sinus infection, sore throat excluding strep throat, asthma and lung disease excluding asthma, respectively) with ref. to absence of condition, adjusted for use of prescribed medications
**Diet**					
**Carbohydrate intake**					
**Karadağ**, ***et al****.***, 2019**	**4596**	**OR 0.73**	**OR 0.61–0.87**	**<0.001**	**Consumption of white rice; comparison and reference groups not specified**
**Karadağ**, ***et al****.***, 2019**	**4596**	**OR 0.66**	**OR 0.53–0.83**	**<0.001**	**Consumption of whole-grain bread; comparison and reference groups not specified**
**Dairy intake**					
**Aalemi, Anwar, & Chen, 2019;**	**558**	**OR 2.36 (whole milk)** **OR 1.95 (low fat milk)**	**OR 1.39–4.01 (whole milk)** **OR 1.10–3.45 (low fat milk)**	**0.002 (whole milk)** **0.021 (low fat milk)**	**Drinking milk (whole or low fat, respectively) ≥ 3 days per week with ref. to drinking milk (whole or low fat, respectively) <3 days per week**
**Di Landro**, ***et al****.***, 2012**	**563**	**OR 1.78**	**OR 1.22–2.59**	**N/A**	**Drinking >3 average daily portions of milk per week with ref. to drinking ≤3 average daily portions of milk per week, OR adjusted for age, BMI and family history**
*Ulvestad, Bjertness, Dalgard & Halvorsen, 2017*	*2489*	*OR 1.56*	*OR 1.02–2.39*	*Not reported*	*≥2 cups full-fat dairy product consumed per day with ref. to no full-fat dairy product consumed, OR adjusted for household income, ethnicity, stress levels and BMI*
*Ulvestad, Bjertness, Dalgard & Halvorsen, 2017*	*2489*	*OR 1.80*	*OR 1.02–3.16*	*Not reported*	*Females who consumed ≥2 cups full-fat dairy product per day with ref. to females who did not consume any full-fat dairy products per day, OR adjusted for household income, ethnicity, stress levels and BMI*
**Egg intake**					
**Aalemi, Anwar, & Chen, 2019;**	**558**	**OR 1.95**	**OR 1.20–3.17**	**0.007**	**Eating eggs ≥ 3 days per week with ref. to eating eggs <3 days per week**
**Fat intake**					
**Aalemi, Anwar, & Chen, 2019;**	**558**	**OR 3.57**	**OR 2.20–5.80**	**<0.001**	**Eating potato chips ≥ 3 days per week with ref. to eating potato chips <3 days per week**
Al Hussein, *et al*., 2016	148	OR 4.091	OR 1.502–11.144	0.0049	>100 g of dietary fat consumed 2–4 times per week with ref. to>100 g of dietary fat consumed less than 2 times per week
Ghodsi, Orawa, & Zouboulis^51^	1002	Not reported	Not reported	0.02	Regular consumption of oily food is correlated with increased acne severity
**Fish intake**					
**Di Landro**, ***et al****.***, 2012**	**563**	**OR 0.68**	**OR 0.47–0.99**	**N/A**	**Eating ≥ 1 average daily portion of fish per week with ref. to eating <1 average daily portion of fish per week, OR adjusted for age, BMI and family history**
**Fruits and vegetables intake**					
Al Hussein, *et al*., 2016	148	OR 0.221	OR 0.068–0.717	0.0131	250 g of fruits and vegetables 2–3 times per day, with ref to 250 g of fruits and vegetables less than 2 times per day
**Karadağ**, ***et al****.***, 2019**	**4596**	**OR 0.71**	**OR 0.57–0.81**	**0.001**	**Consumption of watermelon; comparison and reference groups not specified**
**Karadağ**, ***et al****.***, 2019**	**4596**	**OR 1.30**	**OR 1.05–1.60**	**0.01**	**Consumption of processed fruit juice; comparison and reference groups not specified**
**Meat intake**					
**Aalemi, Anwar, & Chen, 2019;**	**558**	**OR 0.27**	**OR 0.15–0.49**	**<0.001**	**Consuming chicken ≥ 3 days per week with ref. to consuming chicken <3 days per week**
**Nut intake**					
Ghodsi, Orawa, & Zouboulis^51^	1002	Not reported	Not reported	<0.0005	Regular nut consumption is correlated with increased acne severity
**Sugars/chocolates intake**					
**Aalemi, Anwar, & Chen, 2019;**	**558**	**OR 2.19**	**OR 1.36–3.53**	**0.001**	**Eating chocolate ≥ 3 days per week with ref. to eating chocolate <3 days per week**
Al Hussein, *et al*., 2016	148	OR 4.092	OR 1.491–11.233	0.0107	>100 g of sweets per day with ref. to <100 g of sweets per day
Ghodsi, Orawa, & Zouboulis^51^	1002	Not reported	Not reported	0.03 (chocolates) <0.0005 (sweets)	Regular chocolate and sweets consumption, respectively, are correlated with increased acne severity
**Karadağ**, ***et al****.,* **2019**	**4596**	**OR 1.48**	**OR 1.24–1.76**	**<0.001**	**>3 portions of chocolate per week with ref. to ≤ 3 portions of chocolate per week**
**Karadağ**, ***et al****.***, 2019**	**4596**	**OR 0.69**	**OR 0.55–0.87**	**0.002**	**Consumption of cookies; comparison and reference groups not specified**
**BMI**					
Aksu, *et al*., 2012	2300	Not reported	Not reported	0.006	More severe acne was associated with being overweight
Al Hussein, *et al*., 2016	148	OR 5.027	OR 1.284–19.682	0.0210	BMI ≥ 25 (overweight and obese) with ref. to BMI < 25
**Di Landro**, ***et al****.***, 2012**	**563**	**OR 1.90 (BMI 18.5–23)** **OR 1.94 (BMI > 23)**	**OR 1.09–3.31 (BMI 18.5–23)** **OR 1.02–3.68 (BMI > 23)**	**N/A**	**BMI 18.5–23 with ref. to BMI < 18.5 or BMI > 23 with ref. to BMI < 18.5 respectively, OR adjusted for age and family history**
**Karadağ**, ***et al****.***, 2019**	**4596**	**Not reported**	**Not reported**	**<0.001**	**Significant association between acne severity and BMI**
Karciauskiene, Valiukeviciene, Gollnick & Stang, 2014	1229	OR 2.0	OR 1.4–3	N/A	BMI ≥ 25 (overweight and obese) was associated with increased risk of getting mild acne with ref. to BMI < 25 (normal weight), OR adjusted for age and sex
**Lu**, ***et al****.***, 2017**	**659**	**OR 5.027**	**OR 2.758–9.162**	**<0.001**	**Overweight/obese BMI with ref. to underweight/normal BMI**
**Dieting (to lose weight)**					
**Aalemi, Anwar, & Chen, 2019;**	**558**	**OR 0.31**	**OR 0.13–0.74**	**0.009**	**Being on a diet to lose weight in the last year with ref. to not being on a diet to lose weight in the last year**
**Substance Use**					
**Smoking status (cigarettes)**					
**Karadağ**, ***et al****.***, 2019**	**4596**	**Not reported**	**Not reported**	**<0.001**	**Severe acne occurred more frequently in smokers**
Klaz, Kochba, Shohat, Zarka & Brenner, 2006	27083	OR 0.2	OR 0.06–0.63	<0.0001	Smoking 20–30 cigarettes per day wit ref. to smoking 0 cigarettes per day
Klaz, Kochba, Shohat, Zarka & Brenner, 2006	27083	PR 0.703 (calculated)	N/A	0.0078	Prevalence of severe acne in active smokers with ref. to prevalence of severe acne in non-smokers
Rombouts, Nijsten & Lambert, 2006	594	OR 0.47	OR 0.28–0.77	<0.05	Inflammatory lesions in females who smoked ≥ 3 cigarettes per day for 6 months or more with ref. to inflammatory lesions in females who smoked <3 cigarettes per day
Rombouts, Nijsten & Lambert, 2006	594	OR 0.49 (1–9 per day) OR 0.38 (>9 per day)	OR 0.28–0.87 (1–9 per day) OR 0.16–0.88 (>9 per day)	<0.05	Smoking 1–9 or >9 cigarettes per day, respectively, with ref. to smoking 0 cigarettes per day
Rombouts, Nijsten & Lambert, 2006	594	OR 0.35	OR 0.17–0.70	<0.05	Smoking for a period >24 months with ref. to smoking for a period <6 months
Schäfer, Nienhaus, Vieluf, Berger, & Ring, 2001	896	Not reported	Not reported	0.001	Dose-dependent relationship between severity of acne and number of cigarettes smoked per day
**Lifestyle, stress, skincare, environmental factors**					
**Living environment**					
Aksu, *et al*., 2012	2300	Not reported	Not reported	0.000	More severe acne was associated with living in a semi-rural environment
Kaminsky, Florez-White, Bagatin, & Arias, 2019	1384	Not reported	Not reported	<0.05 (significant)	Exposure to chemical substances was associated with the severity of acne
**Karadağ**, *et al*., **2019**	**4596**	**Not reported**	**Not reported**	**<0.001**	**Significant difference in acne severity in different regions of Turkey**
**Makeup usage**					
Kaminsky, Florez-White, Bagatin, & Arixas, 2019	1384	PR 0.265 (calculated)	N/A	<0.05 (significant)	Use of makeup in individuals with severe acne with ref. to use of makeup in individuals with moderate or mild acne
Perera, Peiris, Pathmanathan, Mallawaarachchi, & Karunathilake^52^	140	PR 3.37 (calculated)	N/A	<0.001	Percentage of severe acne cases who used cosmetics frequently with ref. to percentage of mild acne cases who used cosmetics frequently Cosmetic usage showed a significant positive correlation with acne severity (quantified by acne grades) r = 0.452
**Mental stress**					
Ghodsi, Orawa, & Zouboulis^51^	1002	Not reported	Not reported	<0.0005	Mental stress was positively associated with acne severity
Yosipovitch, *et al*.^56^	94	Not reported	Not reported	0.029	Significant positive correlation between acne severity and stress levels (r = 0.23)
**Physical exercise**					
**Aalemi, Anwar, & Chen, 2019;**	**558**	**OR 0.49**	**OR 0.29–0.84**	**0.009**	**Regular physical exercise with ref. to occasional or no physical exercise**

Study design is indicated via text colour. Black text indicates a cross-sectional design, *italic* text indicates a longitudinal design and bold text indicates a case-control design.

Previous reviews have found that severe acne is more common in males compared to females^21^. Most of the articles reviewed in this study are in line with this trend, demonstrating an association between severe acne and being male (Tables 4, 5). One study reported higher severe acne prevalence in females relative to males, for the age group 11 to 13 but not the 14 to 15 or 16 to 17 age groups^23^, which may be, at least in part, due to the earlier onset of acne in females^2^.

**Table 5:** List of risk factors for acne severity analysed in the articles and results obtained for each risk factor.

Factor	Studies showing		
	Significant risk factors associated with less severe acne	Significant risk factors associated with more severe acne	Insignificant factor
**Demographics**			
Age	N/A	Aksu, *et al*., 2012; Bagatin, *et al*., 2014; Ghodsi, Orawa, & Zouboulis^51^; Park, Kwon, Min, Yoon, & Suh, 2015; Silverberg & Silverberg, 2014; Wu, *et al*.^54^;	Tan, Tan, Barkham, Yan, & Zhu^53^
Household income	N/A	N/A	Silverberg & Silverberg, 2014
Number of children in the family	N/A	Silverberg & Silverberg, 2014 (one child at age 16–17)	N/A
Parent’s education level	N/A	Bagatin, *et al*., 2014 (lower education level in parents); Silverberg & Silverberg, 2014 (highest education level in the family at age 14–15)	N/A
Race	N/A	Silverberg & Silverberg, 2014 (Whites at age 14–15)	Bagatin, *et al*., 2014; Silverberg & Silverberg, 2014 (Hispanic origin, race at age 11–13 and 16–17); Tan, Tan, Barkham, Yan, & Zhu^53^;
Sex	**Karadağ**, ***et al****.***, 2019** (female);	Aksu, *et al*., 2012 (male); Kaminsky, Florez-White, Bagatin, & Arias, 2019 (male); **Karadağ**, ***et al****.***, 2019** (male); Rombouts, Nijsten & Lambert, 2006 (male); Silverberg & Silverberg, 2014 (female at age 11–13)	Bagatin, *et al*., 2014; Ghodsi, Orawa, & Zouboulis^51^; Karciauskiene, Valiukeviciene, Gollnick & Stang, 2014; Schäfer, Nienhaus, Vieluf, Berger, & Ring, 2001; Tan, Tan, Barkham, Yan, & Zhu^53^;
**Genetic and/or Hormonal**			
Family history	Karciauskiene, Valiukeviciene, Gollnick & Stang, 2014 (maternal acne, paternal acne and acne in both parents);	**Aalemi, Anwar, & Chen, 2019** (siblings only); Bagatin, *et al*., 2014 (sibling acne); **Di Landro**, ***et al****.***, 2012** (first-degree relatives with acne); Ghodsi, Orawa, & Zouboulis^51^ (parents and siblings; number of family members with acne history); **Karadağ**, ***et al****.***, 2019** (family members not specified); Karciauskiene, Valiukeviciene, Gollnick & Stang, 2014 (maternal acne, paternal acne and acne in both parents); **Wang**, ***et al****.***, 2016** (family members not specified);	Aksu, *et al*., 2012; Al Hussein, *et al*., 2016; Bagatin, *et al*., 2014 (first-degree relatives other than siblings); Kaminsky, Florez-White, Bagatin, & Arias, 2019; Tan, Tan, Barkham, Yan, & Zhu^53^;
High usage of topical and/or systemic drugs to treat acne	N/A	Rombouts, Nijsten & Lambert, 2006;	Kaminsky, Florez-White, Bagatin, & Arias, 2019 (Regular use of acne drugs)
Menstrual characteristics	Ghodsi, Orawa, & Zouboulis^51^ (premenstrual phase)	N/A	**Aalemi, Anwar, & Chen, 2019** (Age of menarche); **Di Landro**, ***et al****.***, 2012** (regularity of menses); Ghodsi, Orawa, & Zouboulis^51^ (age of first menstruation, regularity of menses); Kaminsky, Florez-White, Bagatin, & Arias, 2019 (age at menarche, onset of menopause); Karciauskiene, Valiukeviciene, Gollnick & Stang, 2014 (onset of puberty);
Sebum production	N/A	Yosipovitch, *et al*.^56^	N/A
Skin type	N/A	Aksu, *et al*., 2012 (oily); Ghodsi, Orawa, & Zouboulis^51^ (seborrheic, personal evaluation of skin oiliness)	Ghodsi, Orawa, & Zouboulis^51^ (winter skin)
Skin colour	N/A	N/A	Bagatin, *et al*., 2014
Use of contraceptives (oral)	Rombouts, Nijsten & Lambert, 2006	N/A	**Di Landro**, ***et al****.***, 2012**; Kaminsky, Florez-White, Bagatin, & Arias, 2019 (hormonal contraceptives);
**Medical history**			
Acanthosis Nigricans	N/A	Kaminsky, Florez-White, Bagatin, & Arias, 2019	N/A
Acne characteristics	N/A	Aksu, *et al*., 2012 (long acne duration); Kaminsky, Florez-White, Bagatin, & Arias, 2019 (onset of acne during adolescence)	N/A
Alopecia	N/A	N/A	Kaminsky, Florez-White, Bagatin, & Arias, 2019
Hirsutism	N/A	Kaminsky, Florez-White, Bagatin, & Arias, 2019	N/A
Hyperseborrhea	N/A	Kaminsky, Florez-White, Bagatin, & Arias, 2019	N/A
Gastrointestinal conditions	N/A	Silverberg & Silverberg, 2014(reflux, abdominal pain, nausea, food allergy),	Silverberg & Silverberg, 2014(frequent diarrhoea, intermittent constipation)
Psychological disorders	N/A	Silverberg & Silverberg, 2014(depression, anxiety, ADHD/ADD, insomnia); **Wang**, ***et al****.***, 2016;**	Silverberg & Silverberg, 2014(phobias)
Sinopulmonary disorders	N/A	Silverberg & Silverberg, 2014 (sinus infection, sore throat, asthma, lung disease excluding asthma, non-streptococcal pharyngitis)	Silverberg & Silverberg, 2014(tonsillitis, hay fever, respiratory allergy)
**Diet**			
Diet in general	N/A	N/A	Al Hussein, *et al*., 2016 (diet in general and lack of nutritional information); Kaminsky, Florez-White, Bagatin, & Arias, 2019;
Carbohydrate intake	**Karadağ**, ***et al****.***, 2019** (whole-grain bread); **Karadağ**, ***et al****.***, 2019** (white rice);	N/A	Al Hussein, *et al*., 2016 (white bread); **Di Landro**, ***et al****.***, 2012** (bread/pasta);
Carbonated drink intake	N/A	N/A	Al Hussein, *et al*., 2016;
Egg intake	N/A	**Aalemi, Anwar, & Chen, 2019;**	N/A
Dairy intake	N/A	**Aalemi, Anwar, & Chen, 2019** (whole milk and low fat milk); **Di Landro**, ***et al****.***, 2012** (milk); *Ulvestad, Bjertness, Dalgard & Halvorsen, 2017* (total dairy intake for females and full-fat dairy intake for the whole sample)	Al Hussein, *et al*., 2016; **Di Landro**, ***et al****.***, 2012** (cheese/yoghurt); *Ulvestad, Bjertness, Dalgard & Halvorsen, 2017* (semi-skimmed or skimmed dairy, moderate intake of dairy in males or the whole sample);
Fasting	N/A	N/A	Ghodsi, Orawa, & Zouboulis^51^
Fat intake	N/A	**Aalemi, Anwar, & Chen, 2019** (potato chips); Al Hussein, *et al*., 2016;	N/A
Fish intake	**Di Landro**, ***et al****.***, 2012;**	N/A	Al Hussein, *et al*., 2016;
Fruit and vegetable intake	Al Hussein, *et al*., 2016; **Karadağ**, ***et al****.***, 2019** (intake of watermelon);	**Karadağ**, ***et al****.***, 2019** (high intake of processed fruit juice);	**Aalemi, Anwar, & Chen, 2019** (vegetables only); **Di Landro**, ***et al****.***, 2012;**
Meat intake	**Aalemi, Anwar, & Chen, 2019** (chicken);	N/A	Di Landro, *et al*., 2012 (red and cured meat);
Nut intake	N/A	Ghodsi, Orawa, & Zouboulis^51^	N/A
Oily food intake	N/A	Ghodsi, Orawa, & Zouboulis^51^	N/A
Spicy food intake	N/A	N/A	Ghodsi, Orawa, & Zouboulis^51^
Sugars/chocolate intake	**Karadağ**, ***et al****.***, 2019** (intake of cookies);	**Aalemi, Anwar, & Chen, 2019** (chocolate only); Al Hussein, *et al*., 2016 (sweets only); Ghodsi, Orawa, & Zouboulis^51^; **Karadağ**, ***et al****.***, 2019** (chocolate only);	**Di Landro**, ***et al****.***, 2012** (cakes, milk-free chocolate and sweets);
BMI (overweight/obese)	Karciauskiene, Valiukeviciene, Gollnick & Stang, 2014;	Aksu, *et al*., 2012 (overweight); Al Hussein, *et al*., 2016; **Di Landro**, ***et al****.***, 2012;** **Karadağ**, ***et al****.***, 2019;** **Lu**, ***et al****.***, 2017;**	N/A
Dieting (to lose weight)	**Aalemi, Anwar, & Chen, 2019;**	N/A	N/A
**Substance Use**			
Cannabis use	N/A	N/A	**Aalemi, Anwar, & Chen, 2019;**
Number of cigarettes smoked/day	Klaz, Kochba, Shohat, Zarka & Brenner, 2006 (inverse dose-dependent relationship); Rombouts, Nijsten & Lambert, 2006; Schäfer, Nienhaus, Vieluf, Berger, & Ring, 2001 (dose-dependent relationship);		N/A
Smoking status (cigarettes)	Klaz, Kochba, Shohat, Zarka & Brenner, 2006 (active smokers); Rombouts, Nijsten & Lambert, 2006	**Karadağ**, ***et al****.***, 2019** (smokers);	Al Hussein, *et al*., 2016; Bagatin, *et al*., 2014; **Di Landro**, ***et al****.***, 2012**; Ghodsi, Orawa, & Zouboulis^51^
Smoking duration (cigarettes)	Rombouts, Nijsten & Lambert, 2006	N/A	N/A
Tobacco use	N/A	N/A	Kaminsky, Florez-White, Bagatin, & Arias, 2019
**Environmental Factors**			
Birthplace	N/A	N/A	Silverberg & Silverberg, 2014 (outside the US)
Duration of residence in the US	N/A	N/A	Silverberg & Silverberg, 2014
Living environment	N/A	Aksu, *et al*., 2012 (semi-rural); **Karadağ**, ***et al****.***, 2019** (region in Turkey)	Al Hussein, *et al*., 2016;
Seasons of the year	N/A	N/A	Ghodsi, Orawa, & Zouboulis^51^; Kaminsky, Florez-White, Bagatin, & Arias, 2019 (climate)
Sun exposure	N/A	N/A	Ghodsi, Orawa, & Zouboulis^51^; Kaminsky, Florez-White, Bagatin, & Arias, 2019 (sun exposure and use of sunbeds)
Travel to humid regions	N/A	N/A	Ghodsi, Orawa, & Zouboulis^51^
**Lifestyle, Stress and Skincare**			
Exposure to chemical substances	N/A	Kaminsky, Florez-White, Bagatin, & Arias, 2019	N/A
Frequency of face washing/day	N/A	N/A	Ghodsi, Orawa, & Zouboulis^51^
Makeup usage	Kaminsky, Florez-White, Bagatin, & Arias, 2019	Perera, Peiris, Pathmanathan, Mallawaarachchi, & Karunathilake^52^ (cosmetics)	Ghodsi, Orawa, & Zouboulis^51^ (use of cream powder, emollient creams)
Mental stress	N/A	Ghodsi, Orawa, & Zouboulis^51^; Yosipovitch, *et al*.^56^	N/A
Physical exercise	**Aalemi, Anwar, & Chen, 2019;**	N/A	Ghodsi, Orawa, & Zouboulis^51^
Sleep duration	N/A	N/A	Ghodsi, Orawa, & Zouboulis^51^

Study design is indicated via text colour. Black text indicates a cross-sectional design, *italic* text indicates a longitudinal design and bold text indicates a case-control design.

Other demographic factors were rarely investigated. In contrast to the results found for acne prevalence, parent’s education level and family education level were found to increase the risk of more severe acne. Further, a study reported a higher risk of severe acne in Whites at age 14 to 15 and those who were the only child at age 16 to 17 while household income was not significantly correlated with acne severity^23^.

### Genetic and hormonal factors that influence acne presentation

**Figure 2:**
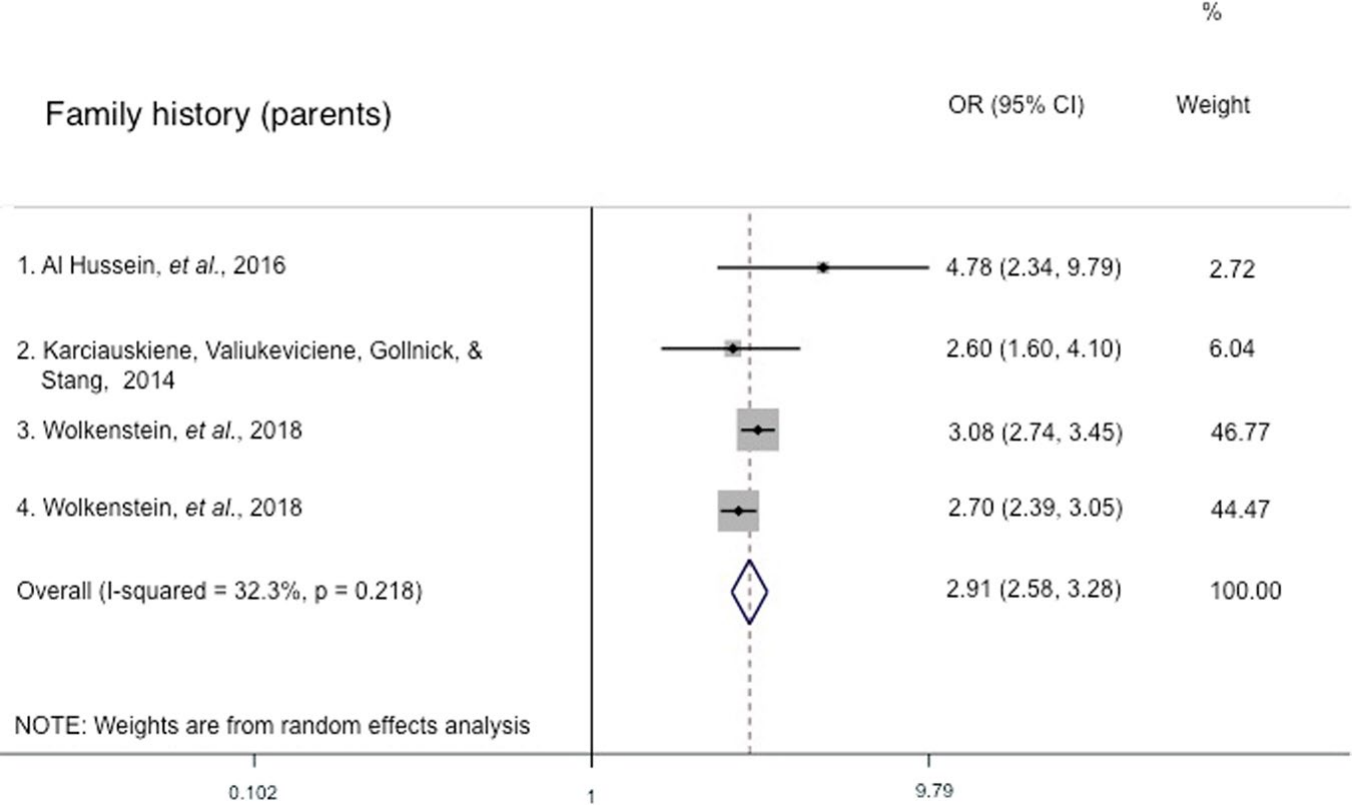
Individual and pooled odds ratio and 95% confidence intervals for acne presentation in association with family history (parents) or no family history (parents). Four studies were excluded from meta-analysis due to a lack of data, such as the odds ratio and/or 95% confidence interval. Five additional studies were excluded from meta-analysis due to study design or because the comparison and reference groups used were different from the other studies. Two entries are used for Wolkenstein *et al*.^49^ as the odds ratios for maternal family history and paternal family history were presented separately.

Studies have also demonstrated the impact of genetic factors on acne presentation. Dreno and Poli^24^ reported that a positive family history of acne in parents was associated with increased acne risk in their offspring. While the articles reviewed in this study used different definitions of family history, with some considering only parents and others including siblings, first- or second-degree relatives, regardless of the definition used, the large majority of the articles consistently found that a positive family history was significantly associated with increased odds of acne in individuals. The pooled odds ratio of 2.91 (95% CI 2.58–3.28; family history in parents with reference to no family history in parents) suggests that family history in parents is associated with an increased risk of acne presentation (Fig. 2). Two case-control studies were excluded from the meta-analysis due to study design, but their results were still in line with the observed trend in the meta-analysis^11,25^. In addition, a study seems to suggest a possible additive effect of maternal and paternal family history on the prevalence of acne, as a larger odds ratio of 2.6 is observed if both parents have acne, compared to an odds ratio of 2.1 and 1.7 for maternal and paternal acne, respectively^26^. However, as few studies present data for maternal, paternal and both parents that allow for such comparison, further studies are required to determine if a true additive effect is present.

Further, an individual’s skin type (for example oily, neutral or dry skin) can be classified according to their skin sebum level. Twin studies suggest that skin sebum levels are controlled by genetic factors^27^. Oily skin shows a strong association while mixed skin shows an association with acne presentation relative to neutral or dry skin (Tables 2, 3). Since *Propionibacterium acnes* favor environments with high sebum levels^19^, having oily and mixed skin characterized by higher sebum levels increases the risk of acne presentation.

Other genetic factors were considered, with one study finding that acne was more prevalent in taller individuals and in those with a lighter skin tone. A few studies also considered factors related to hormones. Contraceptive use and the regularity of menstruation were not significantly linked to acne prevalence while pregnancy status and onset of puberty were associated with acne prevalence, with those who have never been pregnant and post-pubertal individuals at a higher risk of acne.

### Genetic and hormonal factors that influence acne severity

Bhate and Williams^19^ observed that heritability estimates and twin studies suggest a genetic basis for acne and reported that individuals with a family history of acne tend to have more severe acne. In contrast to this observation, the articles reviewed suggest that a positive family history may not necessarily correlate with increased acne severity – approximately half of the articles reviewed found significantly higher odds of severe acne in those with a positive family history while the other half found that family history was not significantly associated with acne severity. A meta-analysis using loose criteria was conducted to study the association of acne severity with positive family history of acne with reference to no family history of acne (Supplementary Fig. S1), and the results suggest that family history may increase the risk of more severe acne. However, this result should be interpreted with caution due to the use of loose meta-analysis criteria. As mentioned earlier, some evidence seems to suggest a possible additive effect of family history on acne presentation. However, this presence or absence of this additive effect may depend on acne severity. A study observed an additive effect for those with mild acne but not for those with severe acne^26^. Further research is needed to determine if there is an interaction between additive effects of family history and acne severity.

Skin type can also influence acne severity. Oily (whether dermatologist- or self-evaluated) and seborrheic skin was observed to be associated severe acne (Tables 4, 5). Similarly, those with more severe acne were more likely to have higher sebum production and high usage of drugs to treat their acne. Most studies also noted that in general, menstrual characteristics (such as onset of puberty and regularity of menses) were not significantly related to acne severity. One study also found that skin color was not associated with acne severity. In addition, oral contraceptives may be protective for more severe acne.

### Dietary factors that influence acne presentation

The importance of dietary factors in influencing acne presentation has been widely debated^28^. In particular, dairy and chocolate intake have received the most attention, possibly due to the hypothesis that Western diets are related to acne. The papers reviewed that studied the influence of the intake of various dairy products – including dairy in general, ice cream, yoghurt, cheese and different types of milk – on the presentation of acne elicited differing results (Tables 2, 3). Most studies found that cheese, yoghurt and ice cream intake did not significantly impact the risk of acne. The influence of milk intake on acne risk, however, was unclear, with inconsistent results between studies. For example, Ismail, Manaf and Azizan^13^ found that drinking milk on at least once per week was linked to increased odds of acne to 3.99 relative to those who drank milk less than once per week while Adebamowo *et al*.^29^ found that the intake of whole milk and low fat milk did not significantly influence the odds of acne. However, a meta-analysis review found that the intake of any amount of dairy in general or any kind of milk, regardless of the fat content (full-fat, whole, low-fat, skim) was linked to increased odds of acne^30^, suggesting that dairy and milk intake are likely to influence acne presentation. Possible explanations for this effect implicate steroid hormones or sugars present in milk^21^. Whey, a protein found in milk, may also be linked to acne presentation. One study reported that 5 healthy males developed acne after taking whey protein concentrates^31^. Similarly, Silverberg^32^ observed 5 patients who displayed acne soon after taking whey protein products. 2 patients who stopped taking whey protein showed good response to acne treatments that they did not respond to when they consumed whey protein. Simonart^31^ suggested a link between whey consumption and increased insulin levels, which in turn activates signaling pathways that eventually contribute to acne development. Notably, whey protein is found not only in milk, but also in some protein supplements used to support muscle building^32^.

The influence of chocolate intake on acne presentation is also the subject of debate^19^. Several studies, regardless of design, found that high or frequent chocolate intake was associated with increased odds of acne presentation (Tables 2, 3). A few hypotheses have been suggested to explain the possible effect of dairy and chocolate consumption on acne presentation. One hypothesis suggests that the sugars in dairy products and chocolate trigger insulin secretion, activating signaling pathways that eventually lead to increased keratinocyte proliferation, which can lead to the formation of acne lesions^21,28^. This may explain why some studies considered the combined effect of sweets and chocolates on acne presentation^10,33^. An alternative explanation suggests that chocolate consumption increases the secretion of inflammatory cytokines by cells and may influence the formation of acne lesions^28^. Meta-analyses using loose criteria were conducted to investigate the association of acne with chocolate intake (Supplementary Fig. S4), and the results suggest that high chocolate intake may increase the risk of acne. However, this result should be interpreted with caution due to the use of loose meta-analysis criteria.

High fat intake may also be a potential dietary risk factor for acne. Despite the inconsistency in the results from different studies, several studies found that high fat intake increases the odds of acne presentation. While potential mechanisms to explain this effect have been suggested, the evidence in support of them is insufficient^34^. More work is needed to establish the relationship between fat intake and acne and understand the possible mechanisms involved. Several dietary factors may be protective for acne. High intake of fish is suggested to reduce the risk of acne^35^, (Tables 2, 3). However, Wang *et al*.^36^ observed that seafood intake was associated with increased risk acne presentation, suggesting that the intake of other types of seafood may be associated with an increased rather than decreased risk of acne. Furthermore, some studies report that a high intake of fruits and vegetables may lower the risk of acne presentation. These observed protective effects may be due to the omega-3 fatty acids found in fish and the high fibre content in fruits and vegetables, which have been shown to lower the levels of Insulin-like growth factor 1 (IGF-1), thus reducing acne risk^37^.

In general, most studies reported that acne prevalence shows no relationship with the intake of carbonated drinks, fast food, meat, vitamins and minerals, nuts, and rice, pasta and semolina. Similarly, irregular mealtimes and a lack of nutritional information were not linked to acne prevalence. In addition, relatively few studies found that high glycemic load, spicy food and white bread was linked to increased risk of acne while a Mediterranean diet was linked to decreased risk of acne. The effect of diet in general and the intake of sugary foods, such as cakes and sweets, on acne is unclear, with several studies suggesting that they are risk factors while other studies found them insignificant. However, since these factors were only investigated in few studies, these results require further verification by future research.

### Dietary factors that influence acne severity

The influence of chocolate intake on acne severity is also the subject of debate^19^. A few studies found that chocolate intake was associated with increased acne severity (Tables 4, 5). However, milk may confound the relationship between chocolate intake and acne severity, since many types of chocolate contain milk^38^. In a clinical study, 99% dark chocolate was used to control for this potential confounder. The study found that the daily intake of a small amount of 99% dark chocolate for four weeks resulted in a statistically significant increase in acne severity grades of participants^38^. However, it is unclear why chocolate intake is associated with more severe acne. One explanation implicates cocoa butter, a component of chocolate that contains high levels of oleic acid. Experiments conducted in animals demonstrated that oleic acid can affect the keratinization of skin and promote the development of comedones^39^. Oleic acid may have a similar effect in humans, promoting the formation of comedones and contributing to more severe acne.

Three studies also suggested that milk intake may increase the risk of more severe acne^40–42^, which is consistent with a meta-analysis that reported that high milk consumption was significantly associated with the presentation of moderate-severe acne^43^. Explanations for the association between milk intake and acne presentation may also explain the association observed for milk intake and acne severity. In addition, to investigate the association of acne severity with milk intake, a meta-analysis using loose criteria was performed (Supplementary Fig. S3), and the results suggest that high milk intake may increase the risk of more severe acne. However, this result should be interpreted with caution due to the use of loose meta-analysis criteria.

Some studies report that high dietary intake of fruits and vegetables may lower the risk of severe acne. However, processed fruit juices consumption does not confer this protective effect, but instead, is associated with increased acne severity^44^. A high dietary intake of fish may be associated with not only reduced acne risk, but also reduced risk of severe acne^41^ (Table 5).

In addition, a few studies found that acne severity was not significantly associated with diet in general, fasting, a lack of nutritional information, carbonated drink intake, bread/pasta intake, cheese/yoghurt intake, red meat intake, cured meat intake and spicy food intake. Intake of nuts, intake of eggs, intake of potato chips, high intake of fat and intake of oily food was linked to more severe acne while intake of whole grain bread, white rice and chicken and dieting to lose weight were linked to less severe acne. However, since these factors were only investigated in a small number of studies, these results need to be further verified by future research.

### Personal factors that influence acne presentation

**Figure 3:**
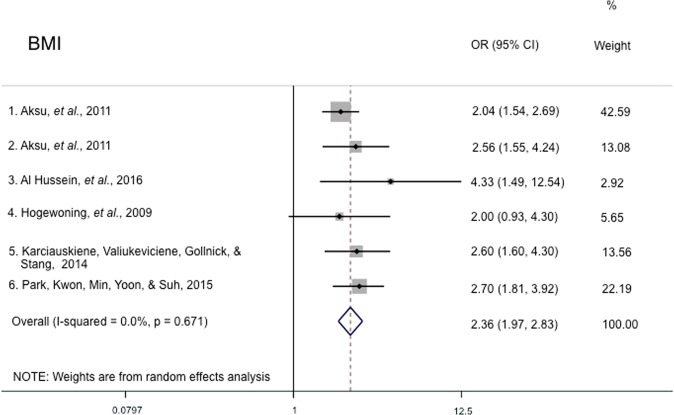
Individual and pooled odds ratio and 95% confidence intervals for acne presentation in association with overweight/obese individuals or normal/underweight individuals. One study was removed from the meta-analysis due to study design. Two entries are used for Aksu *et al*.^48^ as the odds ratio for normal BMI and overweight BMI were presented separately.

Personal factors including Body Mass Index (BMI), smoking status and alcohol intake have previously been linked to acne presentation. Most studies have noted increased prevalence of acne in overweight and obese individuals (typically defined as BMI ≥23 kg/m^2^ and BMI ≥25 kg/m^2^, respectively) relative to underweight individuals (BMI <18.5 kg/m^2^) or individuals of a normal weight (18.5 kg/m^2^ ≤ BMI < 23 kg/m^2^). A pooled odds ratio was calculated to establish the association between BMI and acne risk (Fig. 3). The obtained OR of 2.36 (95% CI 1.97–2.83; overweight/obese BMI with reference to normal/underweight BMI) suggests that BMI significantly influences acne presentation. While a case-control study by Lu *et al*.^45^ was excluded from the meta-analysis because of study design, their results were in line with the trend found in the cross-sectional studies. Obese and overweight individuals tend to have higher glycemic loads and androgen levels, which may increase sebum secretion, promoting the formation of acne lesions^26^. Dietary factors may confound the relationship between BMI and acne.

The effect of smoking status on acne prevalence is controversial, with inconsistent evidence on whether smoking is a protective or risk factor^19^. Rombouts, Nijsten and Lambert^22^ observed that in girls, smoking was linked to decreased odds of acne of 0.41 (95% CI 0.13–0.82) while Al Hussein *et al*.^35^ reported that smoking was a risk factor for acne, increasing the odds of acne to 2.859 (95% CI 1.467–5.576). Studies also considered smoking duration and number of cigarettes smoked. Smoking has been proposed to influence acne through multiple pathways that may affect processes such as inflammation, wound healing and immune responses^22^. For example, nicotine in cigarette smoke is suggested to activate receptors on cells such as keratinocytes, slowing the process wound healing and promoting acne in patients, however, there is a lack of evidence supporting this claim^21^. In contrast, smoking is also suggested to reduce inflammation, protecting against inflammatory acne^22^. The complex way in which smoking influences the pathogenesis of acne may contribute, in part, to the inconsistency in the findings from different studies. Study design may also affect the results obtained in different studies. A meta-analysis found a no significant association between smoking and acne prevalence when all studies were included, however, when studies with a quality score ≤6 were excluded, a significant protective effect of smoking on acne prevalence was observed^46^.

A small number of studies have also investigated the relation between substance use and acne prevalence. Alcohol intake was consistently found to have no significant relation to acne prevalence. Tobacco use may be protective for acne while cannabis use may be linked to increased acne risk. In addition, a few studies found that lack of sleep or insomnia, sun exposure, high mental stress, study pressure and cosmetic usage may be associated with increased acne presentation. In contrast, frequent face washing may be linked to lowered acne risk. One study also found no significant association between acne and both physical exercise sunbed usage. However, some factors show inconsistent results. For example, Hogewoning *et al*.^47^ found that living in an urban environment was linked to increased acne risk, while the findings from Aksu *et al*.^48^ suggest the opposite. For country of residence, living in certain countries, like Poland and Belgium, was linked to reduced acne risk while living in Czech or Slovak Republic was linked to increased acne risk. Further research is necessary to establish the association link and understand possible mechanisms underlying these associations.

### Personal factors that influence acne severity

Previous studies have suggested a link between personal factors and acne severity. Most studies suggest a strong association between overweight or obese BMI and acne severity, with Al Hussein *et al*.^35^ reporting an odds ratio of 5.02 (95% CI 1.284–19.682; BMI ≥25 relative to BMI <25) and Lu *et al*.^45^ reporting a similar odds ratio 5.027 (95% CI 2.758–9.162; overweight/obese BMI relative to underweight/normal BMI). A meta-analysis using loose criteria was conducted to determine the association of acne severity with overweight/obese BMI with reference to normal or underweight BMI (Supplementary Fig. S2), and the results suggest that overweight/obese BMI may increase the risk of more severe acne. However, this result should be interpreted with caution due to the use of loose meta-analysis criteria. Similar mechanisms may explain the association between BMI and both acne presentation and acne severity.

The effect of smoking status on acne severity is controversial. Rombouts, Nijsten and Lambert^22^ observed that in girls, smoking was protective of severe acne while Karadağ *et al*.^44^ found that severe acne occurred more frequently among smokers. The effects of smoking duration and number of cigarettes smoked on acne severity were also investigated in some studies. While one study observed a significant dose-dependent relationship between acne severity and the number of cigarettes smoked per day^17^, other studies did not find a significant dose-dependent effect^22^. Explanations for the association between smoking and acne presentation may also explain the association between smoking and acne severity.

A small number of studies have considered the influence of other factors on acne severity. Makeup use, high mental stress and exposure to chemical substances were shown to be associated with more severe forms of acne while cannabis use, tobacco use, lack of sleep, sun exposure, seasons of the year, climate, travel to humid regions, birthplace, duration of residence in the US and frequent face washing were not significantly associated with acne severity. Living in a semi-rural environment or certain regions in Turkey and physical exercise may be protective for severe acne. Further research is necessary to establish the association link and understand possible mechanisms underlying these associations.

### Limitations and conclusions

When selecting articles for review, no exclusion criteria based on geographical location was used. However, because the selected articles were unable to fully represent all regions and continents in the world (for example, no studies from the Australian continent were included based on the selection criteria), the result may not apply to the areas that were not included in the study. In addition, most of the articles reviewed are cross-sectional or case-control studies where the variables have not been experimentally manipulated, thus we cannot exclude the possibility of confounders that may cloud the true relationship between a factor and acne presentation or severity. Furthermore, despite the use of objective criteria to evaluate potential factors influencing acne presentation and severity, the analysis may be subject to researcher bias. Inadvertent errors may also have occurred when summarizing the data from primary literature.

We conducted meta-analysis on risk factors with replicated results reported in a minimum of three independent acne publications. However, we faced some difficulties as some studies reported incomplete information and failed to report the odds ratio and/or 95% odds ratio confidence interval. In addition, we noted that for each particular factor, the comparison and reference groups used in different studies were not compatible, making it difficult to do a meta-analysis, as the results were not comparable. Further, the different study designs used made it difficult to compare the results of different studies via meta-analysis. Thus, meta-analysis was not conducted on all factors reviewed.

Based on the analysis, the potential factors that are the most likely to influence acne presentation and severity are family history and BMI. However, more work needs to be done on dietary factors, smoking, mental stress and sleep duration to understand their effects on acne presentation and severity.

This study summarizes the potential factors that may affect the risk of acne presentation or severe acne and can help researchers and clinicians to understand the epidemiology of acne and severe acne. In addition, the findings can guide future research on risk factors with the hope of better understanding the pathophysiology of acne and developing effective therapeutics.

## Methods

### Search strategy and selection criteria

**Figure 4:**
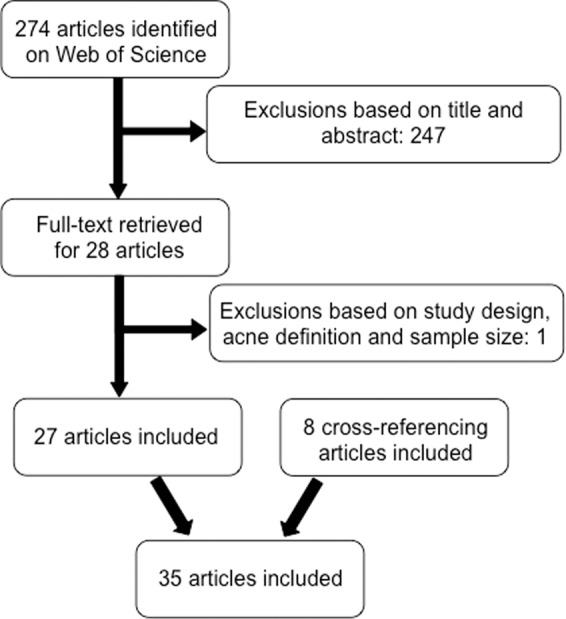
Flowchart of the process used to select studies for meta-analysis.

A search was conducted on the Web of Science database in September 2019 and only papers of the document type ‘article’ published between 1990 and 2019 containing the search term ‘acne’ in the title and the terms ‘epidemiology’ and ‘risk factor’ in the topic were included. ‘Acne’ was used instead of ‘acne vulgaris’ as it is more general and commonly used. 274 articles were identified after this initial search. Since this article is only interested in modifiable risk factors of acne vulgaris, articles that discussed other forms of acne (such as acne rosacea), polymorphisms associated with acne, acne as a risk factor for other psychological or medical conditions, the efficacy of acne treatments and any other irrelevant articles were excluded. One article with an underspecified study design, unclear acne definition and small sample size was also excluded. Unlike other studies that determined acne status via self-report or clinical evaluation, no indication was made as to how acne status was determined in this particular study, leading to exclusion. Based on these criteria, 247 articles were excluded from analysis. A total of 35 articles – 27 articles from the described search and 8 additional articles for cross-referencing – were analyzed carefully for study design, acne prevalence, acne definition, acne severity grading system used and risk factors evaluated. The process followed to select studies for review is shown in Fig. 4.

### Meta-analysis

Stata/MP 14.0 was used to conduct meta-analysis using the random effects model to investigate the effect of risk factors with replicated results reported in a minimum of three independent acne publications. Studies with incomplete information such as those that failed to report the 95% odds ratio confidence interval or those that did not clearly define the reference and comparison groups used to calculate the odds ratio were excluded from the meta-analysis. Further, only the results from studies that used a similar design (in this case, cross-sectional design) were included in the meta-analysis.

For meta-analysis using loose criteria, results from studies with different study designs (cross-sectional, case-control and longitudinal) and results that used similar (but not identical) comparison and reference groups were included.

## Supplementary information

Supplementary information.

## References

[1] Vos T (2012). Years lived with disability (YLDs) for 1160 sequelae of 289 diseases and injuries 1990–2010: a systematic analysis for the Global Burden of Disease Study 2010. The Lancet..

[2] Stathakis V, Kilkenny M, Marks R (1997). Descriptive epidemiology of acne vulgaris in the community. Australas J Dermatol..

[3] Williams HC, Dellavalle RP, Garner S (2012). Acne vulgaris. The Lancet..

[4] Mahto A (2017). Acne vulgaris. Medicine..

[5] Cunliffe WJ (1986). Acne and unemployment. Br J Dermatol..

[6] Motley RJ, Finlay AY (1989). How much disability is caused by acne?. Clin Exp Dermatol..

[7] Radtke MA, Schafer I, Augustin M (2010). Pharmacoeconomy in acne–evaluation of benefit and economics. J Dtsch Dermatol Ges..

[8] Wei B (2010). The epidemiology of adolescent acne in North East China. J Eur Acad Dermatol Venereol..

[9] Kaminsky A, Florez-White M, Bagatin E, Arias MI (2019). Iberian Latin American Acne Studies G. Large prospective study on adult acne in Latin America and the Iberian Peninsula: risk factors, demographics, and clinical characteristics. Int J Dermatol..

[10] Park SY, Kwon HH, Min S, Yoon JY, Suh DH (2015). Epidemiology and risk factors of childhood acne in Korea: a cross-sectional community based study. Clin Exp Dermatol..

[11] Di Landro A (2016). Adult female acne and associated risk factors: Results of a multicenter case-control study in Italy. J Am Acad Dermatol..

[12] Pereira Duquia R (2017). Epidemiology of Acne Vulgaris in 18-Year-Old Male Army Conscripts in a South Brazilian City. Dermatology..

[13] Ismail NH, Manaf ZA, Azizan NZ (2012). High glycemic load diet, milk and ice cream consumption are related to acne vulgaris in Malaysian young adults: a case control study. BMC Dermatology..

[14] Klaz I, Kochba I, Shohat T, Zarka S, Brenner S (2006). Severe acne vulgaris and tobacco smoking in young men. J Invest Dermatol..

[15] White GM (1998). Recent findings in the epidemiologic evidence, classification, and subtypes of acne vulgaris. J Am Acad Dermatol..

[16] Tan J (2012). Acne severity grading: determining essential clinical components and features using a Delphi consensus. J Am Acad Dermatol..

[17] Schäfer T, Nienhaus A, Vieluf D, Berger J, Ring J (2001). Epidemiology of acne in the general population: the risk of smoking. Br J Dermatol..

[18] Bagatin E (2014). Acne vulgaris: prevalence and clinical forms in adolescents from Sao Paulo, Brazil. An Bras Dermatol..

[19] Bhate K, Williams HC (2013). Epidemiology of acne vulgaris. Br J Dermatol..

[20] Janani S, Sureshkumar R (2019). A Comprehensive Review on Acne, its Pathogenesis, Treatment, *In-Vitro* and *In-Vivo* Models for Induction and Evaluation Methods. Int J Pharm Sci.

[21] Lynn DD, Umari T, Dunnick CA, Dellavalle RP (2016). The epidemiology of acne vulgaris in late adolescence. Adolesc Health Med Ther..

[22] Rombouts S, Nijsten T, Lambert J (2007). Cigarette smoking and acne in adolescents: results from a cross-sectional study. J Eur Acad Dermatol Venereol..

[23] Silverberg JI, Silverberg NB (2014). Epidemiology and extracutaneous comorbidities of severe acne in adolescence: a U.S. population-based study. Br J Dermatol..

[24] Dreno B, Poli F (2003). Epidemiology of acne. Dermatology..

[25] Goulden V, McGeown CH, Cunliffe WJ (1999). The familial risk of adult acne: a comparison between first-degree relatives of affected and unaffected individuals. Br J Dermatol..

[26] Karciauskiene J, Valiukeviciene S, Gollnick H, Stang A (2014). The prevalence and risk factors of adolescent acne among schoolchildren in Lithuania: a cross-sectional study. J Eur Acad Dermatol Venereol..

[27] Walton S, Wyatt EH, Cunliffe WJ (1988). Genetic control of sebum excretion and acne—a twin study. Br J Dermatol..

[28] Tan JK, Bhate K (2015). A global perspective on the epidemiology of acne. Br J Dermatol..

[29] Adebamowo CA (2008). Milk consumption and acne in teenaged boys. J Am Acad Dermatol..

[30] Juhl, C. R. *et al*. Dairy Intake and Acne Vulgaris: A Systematic Review and Meta-Analysis of 78,529 Children, Adolescents, and Young Adults. *Nutrients*.;10(8), 10.3390/nu10081049 (2018). 10.3390/nu10081049 PMC6115795 30096883

[31] Simonart T (2012). Acne and whey protein supplementation among bodybuilders. Dermatology..

[32] Silverberg NB (2012). Whey protein precipitating moderate to severe acne flares in 5 teenaged athletes. Cutis..

[33] Wolkenstein P (2015). Smoking and dietary factors associated with moderate-to-severe acne in French adolescents and young adults: results of a survey using a representative sample. Dermatology..

[34] Wolf R, Matz H, Orion E (2004). Acne and diet. Clin Dermatol..

[35] Al Hussein SM (2016). Diet, Smoking and Family History as Potential Risk Factors in Acne Vulgaris – a Community-Based Study. Acta Medica Marisiensis..

[36] Wang P (2016). Risk factors, psychological impacts and current treatments of acne in Shanghai area of China. J Dermatolog Treat..

[37] Spencer EH, Ferdowsian HR, Barnard ND (2009). Diet and acne: a review of the evidence. Int J Dermatol..

[38] Vongraviopap S, Asawanonda P (2016). Dark chocolate exacerbates acne. Int J Dermatol..

[39] Choi EH, Ahn SK, Lee SH (1997). The changes of stratum corneum interstices and calcium distribution of follicular epithelium of experimentally induced comedones (EIC) by oleic acid*. Experimental Dermatology..

[40] Aalemi AK, Anwar I, Chen H (2019). Dairy consumption and acne: a case control study in Kabul, Afghanistan. Clin Cosmet Investig Dermatol..

[41] Di Landro A (2012). Family history, body mass index, selected dietary factors, menstrual history, and risk of moderate to severe acne in adolescents and young adults. J Am Acad Dermatol..

[42] Ulvestad M, Bjertness E, Dalgard F, Halvorsen JA (2017). Acne and dairy products in adolescence: results from a Norwegian longitudinal study. J Eur Acad Dermatol Venereol..

[43] Dai R, Hua W, Chen W, Xiong L, Li L (2018). The effect of milk consumption on acne: a meta-analysis of observational studies. J Eur Acad Dermatol Venereol..

[44] Karadag AS (2019). The effect of personal, familial, and environmental characteristics on acne vulgaris: a prospective, multicenter, case controlled study. G Ital Dermatol Venereol..

[45] Lu L (2017). Obese/overweight and the risk of acne vulgaris in Chinese adolescents and young adults. Hong Kong J Dermatol Venereol..

[46] Mannocci A, Semyonov L, Saulle R, Boccia A (2010). Evaluation of the association between acne and smoking: Systematic review and meta-analysis of cross-sectional studies. Ital J Public Health..

[47] Hogewoning AA (2009). Prevalence and risk factors of inflammatory acne vulgaris in rural and urban Ghanaian schoolchildren. Br J Dermatol..

[48] Aksu AE (2012). Acne: prevalence and relationship with dietary habits in Eskisehir, Turkey. J Eur Acad Dermatol Venereol..

[49] Wolkenstein P (2018). Acne prevalence and associations with lifestyle: a cross-sectional online survey of adolescents/young adults in 7 European countries. J Eur Acad Dermatol Venereol..

[50] Ali, F. Determination of various risk factors associated with acne vulgaris infection in Quetta, Pakistan. *Pure and Applied Biology*. **8**(3), 10.19045/bspab.2019.80135 (2019).

[51] Ghodsi SZ, Orawa H, Zouboulis CC (2009). Prevalence, severity, and severity risk factors of acne in high school pupils: a community-based study. J Invest Dermatol..

[52] Perera MPN, Peiris W, Pathmanathan D, Mallawaarachchi S, Karunathilake IM (2018). Relationship between acne vulgaris and cosmetic usage in Sri Lankan urban adolescent females. J Cosmet Dermatol..

[53] Tan HH, Tan AW, Barkham T, Yan XY, Zhu M (2007). Community-based study of acne vulgaris in adolescents in Singapore. Br J Dermatol..

[54] Wu TQ (2007). Prevalence and risk factors of facial acne vulgaris among Chinese adolescents. Int J Adolesc Med Health..

[55] Xu SX (2007). The familial risk of acne vulgaris in Chinese Hans - a case-control study. J Eur Acad Dermatol Venereol..

[56] Yosipovitch G (2007). Study of psychological stress, sebum production and acne vulgaris in adolescents. Acta Derm Venereol..

[57] Ibrahim AA, Salem RM, El-Shimi OS, Baghdady SMA, Hussein S (2019). IL1A (−889) gene polymorphism is associated with the effect of diet as a risk factor in Acne Vulgaris. J Cosmet Dermatol..

[58] Skroza N (2012). Mediterranean diet and familial dysmetabolism as factors influencing the development of acne. Scand J Public Health..

[59] Suppiah TSS (2018). Acne vulgaris and its association with dietary intake: a Malaysian perspective. Asia Pac J Clin Nutr..

