# Supplementary material for: Systematic review of the epidemiology of acne vulgaris
Source: Sci Rep. 2020 Apr 1;10:5754. doi: 10.1038/s41598-020-62715-3 (PMC7113252; doi:10.1038/s41598-020-62715-3)
Supplement: Supplementary file 1 — Supplementary information. [file 41598_2020_62715_MOESM1_ESM.docx]

**Title: Systematic review of the epidemiology of acne vulgaris**

Authors: Anna Hwee Sing HENG^, Fook Tim CHEW^*

**Supplementary Information**

As mentioned in the limitations section of the main paper, we were unable to do a meta-analysis on all factors reviewed due to incomplete information, incompatible comparison and reference groups used in different studies and different study designs used. In this section, meta-analyses have been conducted using looser criteria. We included results from studies with different study designs and results from different studies with similar (but not identical) comparison and reference groups. Because of the use of less stringent criteria, the results presented in this section should interpreted with caution and should be compared with results presented in other studies before coming to a conclusion.

However, meta-analysis still could not be conducted for some factors due to incomplete information, because the factors were studied in less than three independent acne publications, or because the comparison and reference groups used in different studies were too different to be compared.

**Meta-analyses on factors associated with acne severity**

A pooled odds ratio was calculated for the association of acne severity with family history with reference to no family history. Studies that investigated family history in siblings only (Aalemi, Anwar, & Chen, 2019; Bagatin, *et al.*, 2014), parents only (Karciauskiene, Valiukeviciene, Gollnick & Stang, 2014), both parents and siblings (Ghodsi, Orawa, & Zouboulis, 2009), and first-degree relatives (Di Landro, *et al.*, 2012) were included in the meta-analysis. Further, studies that used case-control design and cross-sectional design were included. We observed that a positive family history, regardless of family members studied, was associated with increased risk of more severe acne and the pooled odds ratio obtained was 2.83 (95% CI: 1.86-4.29).

Similarly, a meta-analysis was conducted on the association of acne severity with overweight/obese BMI with reference to normal or underweight BMI. The three studies used slightly different BMI categories in their analysis and we included studies that used case-control design and cross-sectional design. The pooled odds ratio obtained was 3.44 (95% CI: 1.68, 7.02).

**
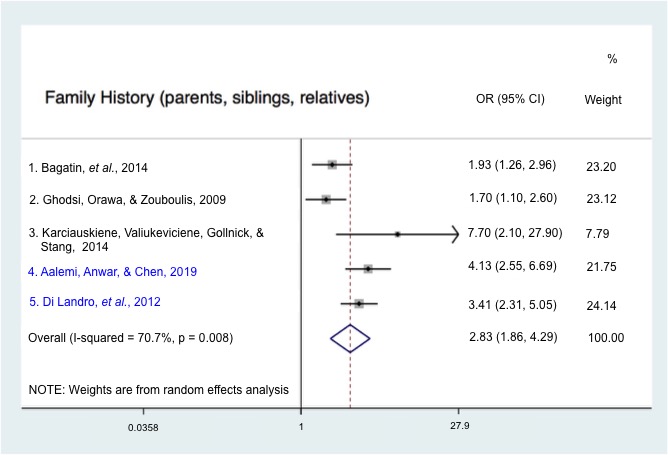
**

**Supplementary Fig. S1** Individual and pooled odds ratio and 95% confidence intervals for acne severity in association with family history or no family history. Study design is indicated via text color. Black text indicates a cross-sectional design, grey text indicates a longitudinal design and blue text indicates a case-control design.


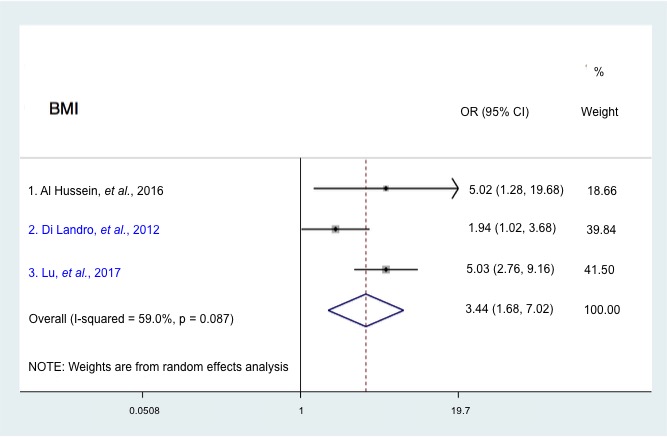


**Supplementary Fig. S2** Individual and pooled odds ratio and 95% confidence intervals for acne severity in association with overweight/obese BMI with reference to normal or underweight BMI. The BMI categories used differed in the three studies. Study design is indicated via text color. Black text indicates a cross-sectional design, grey text indicates a longitudinal design and blue text indicates a case-control design.

The last meta-analysis was performed to investigate the association between acne severity and higher milk intake with reference to lower milk intake. The type of milk studied differed in the three studies: Aalemi, Anwar, & Chen, 2019 studied whole and low fat milk, Di Landro, et al., 2012 studied milk in general and Ulvestad, Bjertness, Dalgard & Halvorsen, 2017 studied full fat dairy intake. Further, the comparison and reference groups used differed in the three studies (Table 5). The calculated pooled odds ratio was 1.83 (95% CI: 1.46, 2.30).


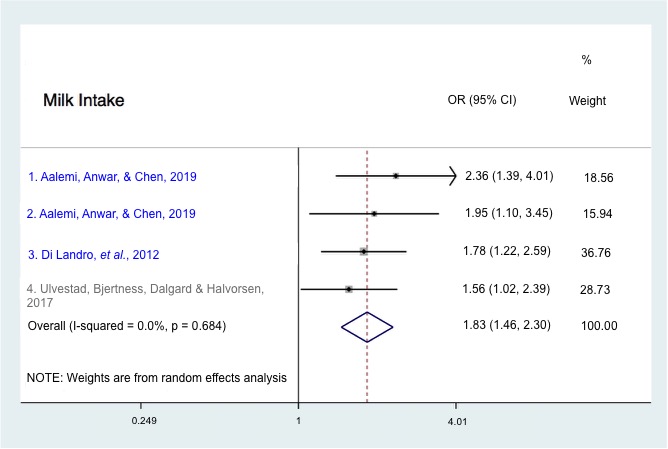


**Supplementary Fig. S3** Individual and pooled odds ratio and 95% confidence intervals for acne severity in association with higher milk intake with reference to lower milk intake. The type of milk studied differed in the three studies. Two entries were used for Aalemi, Anwar, & Chen, 2019 as the odds ratios for whole milk and low fat milk were presented separately. Study design is indicated via text color. Black text indicates a cross-sectional design, grey text indicates a longitudinal design and blue text indicates a case-control design.

**Meta-analyses on factors associated with acne presentation**

A meta-analysis that included case-control and cross-sectional studies was conducted to investigate the association between chocolate intake and acne. The three studies used different comparison and reference groups to calculate the odds ratios. Wolkenstein, *et al*., 2015 compared those who consumed chocolates daily with those who did not consume chocolate, Wolkenstein, *et al.*, 2018 compared quartile 2, 3 or 4 of chocolate consumption, respectively, with quartile 1 of chocolate consumption and Suppiah, *et al.*, 2018 compared those who ate chocolate often to those who seldom ate chocolate. The pooled odds ratio obtained was 1.34 (95% CI: 1.20-1.51), which suggests that higher or more frequent chocolate intake may be associated with increased odds of acne.


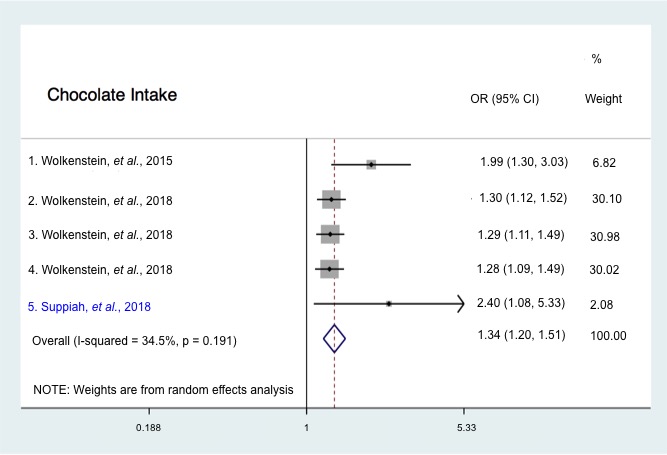


**Supplementary Fig. S4** Individual and pooled odds ratio and 95% confidence intervals for acne presentation in association with higher or more frequent chocolate intake with reference lower or less frequent chocolate intake. Three entries were included for Wolkenstein, *et al.*, 2018 as the odds ratios for quartile 2, 3 and 4 of chocolate consumption were presented separately. Study design is indicated via text color. Black text indicates a cross-sectional design, grey text indicates a longitudinal design and blue text indicates a case-control design.
